# A continuous *in silico* learning strategy to identify safety liabilities in compounds used in the leather and textile industry

**DOI:** 10.1007/s00204-023-03459-7

**Published:** 2023-02-12

**Authors:** Eric March-Vila, Giacomo Ferretti, Emma Terricabras, Inés Ardao, José Manuel Brea, María José Varela, Álvaro Arana, Juan Andrés Rubiolo, Ferran Sanz, María Isabel Loza, Laura Sánchez, Héctor Alonso, Manuel Pastor

**Affiliations:** 1 Department of Medicine and Life Sciences, Research Programme on Biomedical Informatics (GRIB), Hospital del Mar Medical Research Institute (IMIM), Universitat Pompeu Fabra, Barcelona, Spain; 2 Department of Pharmacology, Pharmacy and Pharmaceutical Technology, Innopharma Drug Screening and Pharmacogenomics Platform. BioFarma Research Group. Center for Research in Molecular Medicine and Chronic Diseases (CiMUS), University of Santiago de Compostela, Santiago de Compostela, Spain; 3 Department of Zoology, Genetics and Physical Anthropology, Universidad de Santiago de Compostela, Campus de Lugo, 27002 Lugo, Spain; 4 Preclinical Animal Models Group, Health Research Institute of Santiago de Compostela (IDIS), 15782 Santiago de Compostela, Spain; 5 Department of Sustainability, INDITEX, Av. da Deputación, 15412 Arteixo, Spain

**Keywords:** *In silico*, QSAR, Read across, Leather and textile industry, Computational toxicology, Machine learning

## Abstract

There is a widely recognized need to reduce human activity's impact on the environment. Many industries of the leather and textile sector (LTI), being aware of producing a significant amount of residues (Keßler et al. 2021; Liu et al. 2021), are adopting measures to reduce the impact of their processes on the environment, starting with a more comprehensive characterization of the chemical risk associated with the substances commonly used in LTI. The present work contributes to these efforts by compiling and toxicologically annotating the substances used in LTI, supporting a continuous learning strategy for characterizing their chemical safety. This strategy combines data collection from public sources, experimental methods and *in silico* predictions for characterizing four different endpoints: CMR, ED, PBT, and vPvB. We present the results of a prospective validation exercise in which we confirm that *in silico* methods can produce reasonably good hazard estimations and fill knowledge gaps in the LTI chemical space. The proposed protocol can speed the process and optimize the use of resources including the lives of experimental animals, contributing to identifying potentially harmful substances and their possible replacement by safer alternatives, thus reducing the environmental footprint and impact on human health.

**Supplementary Information:**

The online version contains supplementary material available at 10.1007/s00204-023-03459-7.

## Introduction

In the context of the quest for a circular, sustainable economy, many industries are moving towards a more responsible attitude considering the impact of their processes on the environment and the health of their workers (Cannon 2020; Schroeder et al. 2019; Scoones 2016). In the leather and textile industry (LTI), this change will require a large effort for the comprehensive identification of substances used in the manufacture and the detailed characterization of the toxicological risk associated with each one. At the European level, there are ongoing efforts to define a new chemical strategy (CS)(Conto 2021), in which there is an active participation of the different industrial sectors concerned, such is the case of LTI.

With respect to the first step, a full catalogue of the substances used in LTI has not been completed. Efforts have been made by the Chemsec Textile Guide (2022), compiled by the NGO Chemsec, but it only includes a small number of all the substances used in the LTI. The number of substances used in this industry has been estimated to be 10,000 (Drumond Chequer et al. 2013). From processing natural fibres to the synthesis of polymers, from the dyeing of fabrics to the tanning of leather, many steps involved in clothes manufacturing use thousands of chemical substances. In our globalized world, it is common that manufacturing steps are carried out in distant parts of the globe by providers of fibres, fabrics, dyes, or leather with different degrees of control on the substances involved in their process.

Regarding the characterization of the chemical risk associated with the use of these substances, in many cases, their toxicological information is not complete (Hartung 2009a, b). This does not mean that the industry is not complying with all legislation applicable to its activity, but moving towards a more responsible manufacturing process should go beyond current legal requirements with the aim of identifying potentially harmful substances and progressing in their replacement by safer alternatives.

Even assuming a very conservative estimate, the number of substances used in LTI that are not fully evaluated from a toxicological point of view can amount to more than 10,000 (Drumond Chequer et al. 2013; Keßler et al. 2021; Liu et al. 2021). A complete chemical risk evaluation of every substance would require an enormous investment of time and money. Furthermore, and despite the commitment of the European Union to the Replacement, Reduction and Refinement of testing on animals (Directive 2010/63/EU), if such evaluation is carried out using currently accepted methodologies, it will require an ethically unacceptable number of in vivo experiments.

For this reason, we consider that any strategy for improving the characterization of the chemical risk of these substances must make efficient use of existing knowledge and apply, as much as possible, new approach methodologies (NAM). In particular, *in silico* methods offer the possibility to characterize relevant endpoints with little investment of time and money and they can be applied to a large fraction of the uncharacterized substances used in the LTI to obtain useful estimations of their toxicological properties.

In the present manuscript, we report the first results of a project funded by Inditex, one of the leaders of the LTI, aiming to design a long-time strategy for the characterization of the chemical hazard of substances used in LTI. The ultimate goals of this project are aligned with the Roadmap to Zero (ZDHC, https://www.roadmaptozero.com).

The key aspects of this project are the following:

- Compilation of a comprehensive catalogue of substances used in LTI.
- Annotation of the compounds in this catalogue with relevant toxicological information extracted from public sources.
- Use existing knowledge to fill data gaps by applying computational modelling methods.
- Prospective validation of the annotations and predictions using experimental techniques (most of them approved by OECD).
- Development and implementation of a strategy for the continuous and automatic updating of the LTI-DB.

In a first stage, the chemical risk assessment has been focused on a set of highly relevant toxicological endpoints. With respect to the human health effects, we studied liabilities represented by CMR (carcinogenic, mutagenic, and reprotoxic compounds) and ER (endocrine disruptors) labelling. With respect to ecotoxicity, we focused on the PBT (persistent, bioaccumulative and toxic) and the vPvB (very persistent and very bioaccumulative) labelling.

The project started in 2019, and so far, we have been able to compile a database containing safety information for more than 4634 compounds from 12 open access data sources. Integrated annotations for the four considered endpoints were obtained using decision workflows. Missing annotations were partially covered using ad hoc developed *in silico* models, allowing to predict about 6.483 properties not extracted from the data sources. These predictions were validated by testing their agreement with newly performed experimental results for 32 compounds, carefully selected to represent different reliability scenarios.

The preliminary results, which will be described in detail in the following sections, are only the first step of a long-term strategy, since we plan to keep collecting and curating data, improving our data extraction workflows and maintaining the *in silico* models to generate predictions of growing quality.

## Materials and methods

### Database development

The databases used in the project were developed in PostgreSQL, version 9.5.4, and hosted in a Linux server (2.6.32–642.4.2.el6.x86_64). The chemical information was handled using RDKit (Landrum 2006) version 2019.03.1. Two different databases were generated: Reference Compounds (Compuestos de Referencia, CR) and Compounds of Interest for Inditex (Compuestos de Interés para Inditex, CII). Both stemmed from generic PostgreSQL databases designed to host chemical compounds with biological annotations previously developed in our group. In the case of CR, we used the original schema, while for CII the schema was edited to accommodate the information from the legacy data table. These data were imported into CII using an ad hoc Python script (https://github.com/phi-grib/Itex_codes/tree/master/CreateDB).

When confronted with the problem of generating unique identifiers for all the substances, we decided to use the CAS-RN as the primary database index. This has the advantage of being commonly used in the toxicology field and the related legislation, as well as being accessible for all compounds (no unpublished compounds are referenced). Alternatives based on the chemical structure (e.g. InChi and InChiKey) were tested and considered unsuitable for this use since no chemical structure can be identified for a large portion of the substances of interest (mixtures, extracts, proprietary substances).

### Database compound update

The database update is performed using a semi-automated workflow, consisting of scripts written in Python (https://github.com/phi-grib/Itex_codes_refactorized/blob/master/UpdateDB_ref/Update_CII.py) and in Java which extracts experimental annotations from external sources like ECHA (ECHA 2021), Pharos (Pharos 2021), or PubChem (Kim et al. 2021). ECHA and Pharos are the main sources of annotations, while PubChem is mainly used for obtaining structures from CAS-RN codes when they are not available from alternative sources. The substances collected in our database had hazard annotations obtained from different data sources (Table 1). Initially, only hazard annotations from ECHA and all its subcategories (REACH, CLP…) were included, but Pharos hazard annotations were included in the later versions.

**Table 1:** Data sources

ECHA	Links	Other regulations	Links
EC/1907/2006 (REACH Regulation)	https://echa.europa.eu/regulations/reach/understanding-reach	The Chemical Products (Handling, Import and Export Prohibitions) Ordinance	https://chemycal.com/dap/files/KEMI_Chemical_Products_ordinance-98_944.pdf
EC/1272/2008 (CLP Regulation)	https://echa.europa.eu/regulations/clp/understanding-clp	KEMI Reports 3/25 and 6/15	https://www.kemi.se/en/publications/reports
EC/850/2004 (POPs Regulation)	https://echa.europa.eu/understanding-pops	German Commodity Ordinance (Bedarfsgegenständeverordnung)	https://www.gesetze-im-internet.de/bedggstv/BJNR008660992.html
2000/60/CE (WFD)	https://echa.europa.eu/understanding-wfd	Regulation on prohibitions and restrictions on the marketing and placing on the market of certain substances, mixtures, and products according to the Chemicals Act (Chemikalien-Verbotsverordnung -ChemVerbotsV)	https://www.ecolex.org/details/legislation/chemicals-prohibition-ordinance-lex-faoc167902/
PBT / vPvB Classification (from REACH)	https://echa.europa.eu/understanding-pbt-assessment	EPA Genetox	https://www.nlm.nih.gov/databases/download/genetox.html
Endocrine Disruptors Classification	https://echa.europa.eu/understanding-ed-assessment	Pharos	https://pharosproject.net/

CII database started incorporating an initial set of 3464 substances that belonged to the LTI space. To enlarge this collection, we retrieved compounds and analysed their functional data from diverse sources: associated REACH descriptors were retrieved from the ECHA database, and descriptive unstructured texts were retrieved from the ECHA and Pharos databases. To get the final list of compounds identified as "used in the leather and textile industry", we selected all substances with at least one REACH descriptor that indicated a high probability of a substance being used in LTI.

### Database hazard annotation update

Annotations were obtained from the different ECHA subcategories and introduced in CII. Also, hazard annotations were retrieved from Pharos with an ad hoc crawler and inserted in CII. This process resulted in the addition of 1531 ECHA annotations and 563 Pharos hazard annotations.

### Chemical structure curation

LTI industry uses many substances that cannot be processed by standard cheminformatics tools, like small inorganic substances, organometallics, or complex mixtures. To facilitate the handling of such compounds, we developed Data curation (https://github.com/phi-grib/Data_curation), a Python library, based on RDKit (Landrum 2006) and inspired by the work of Gadaleta et al. (2018).

This tool applies a set of rules on compound importing to identify potential issues and classify input structures as organic, organic salt, organometallic, peptide, inorganic, inorganic salt, inorganic metal, no-sanitizable organic, no-sanitizable inorganic and no-sanitizable organometallic. Substances classified as organometallic, inorganic, inorganic salt, inorganic metal, and no-sanitizable were discarded to avoid errors in the development of *in silico* models.

### Database endpoint annotations

We developed an automatic workflow that uses the hazard annotations extracted from the sources listed in Table 1 to generate an internal integrated assessment for each of the aforementioned endpoints (CMR, ER, PBT, and vPvB). This workflow classifies every substance in CII as "YES" (positive), "NO" (negative), "Pending" (the substance is still in a classification process), or “No Information” for these endpoints. The annotations obtained from this workflow results were stored in CII.

This workflow is depicted in Figure S1, but in a few words, it starts checking ECHA. If the hazard annotations are found under CLP, REACH, and SVHC, a YES is assigned for that given endpoint. Pending is assigned if the annotation is found in other sources like the Registration dossier. Finally, when no hazard annotation is detected, No Information is assigned. Then, our CR database is checked for any annotation that we could have missed and added to CII. The final step was to check in Pharos the hazard annotations for these endpoints, simply translating positives as YES and negatives as NO. If no hazard annotation is found, the compound is annotated as No Information.

Since we are using different sources, we prioritised the ECHA hazard annotations and the positive ones (YES) in the case of inconsistencies. This is a conservative approach, meaning that any positive hazard annotation in an ECHA source was considered enough to annotate the substance as positive for this endpoint.

### Predictive models

*In silico* predictive models were generated using the following protocol.

#### Data preparation

CII was queried to extract all substances as a table of names, SMILES, and the internally generated annotations (see Database endpoint annotations section). Annotations were all qualitative (YES, NO, No Information). Only compounds with YES (coded as 1) or NO (coded as 0) were used for the modelling.

In the case of CMR, Pending was considered positive (YES) as well. We followed a conservative approach: if something could be CMR, we took it as CMR. Substances in the No Information category were not used for model building, only for prediction. Problematic structures, including organometallics, inorganic compounds, or salts, were removed.

In most cases, the datasets extracted using this method were highly imbalanced, with many more positive than negative compounds. This is a well-studied problem that affects the quality and predictive performance of predictive models (Stewart, 2020). Imbalance correction algorithms (Lemaître et al. 2017) were applied to mitigate its effect. When this protocol successfully obtains a reasonably balanced dataset, a classifier is used to build a qualitative QSAR model, as explained in the QSAR modelling section. Otherwise, when the number of compounds present in one class was too small, we used read-across (RAX) as a last resource.

The datasets obtained were the following:

- PBT and vPvB sets: We generated training sets, applying oversampling to the positive class. These sets contained only annotated compounds from CII (YES and NO). The prediction sets contained all the compounds of the database with the problematic structures removed. The number of substances for each class is in Table 2.
- CMR set: No imbalance correction algorithm produced a model of enough quality in this case. To obtain well-balanced datasets, while preserving all the information in the original data, three different balanced datasets were generated by including all negative compounds and a randomly selected sample of positive compounds of the same size. At the end, all the positives were included in at least one of the datasets. These series were used to build separate models, which were then combined in an ensemble model, as described in the QSAR modelling section. The prediction set again contained all the compounds without the problematic structures. Table 2 contains the number of substances for each set and the class proportions.
- ED set: In this case, the number of negative compounds (only three) was too low to attempt any unbalance correction. As a contingency solution, we applied a RAX analogue approach, as described in the RAX section. The number of positive compounds is shown in Table 3.

**Table 2:** Sizes of PBT, vPvB and CMR datasets, after resampling

Endpoint	Molecules	Negative class	Positive class
PBT	240	187	53
vPvB	254	189	65
CMR ensemble	596	285	311
CMR set 1	494	247	247
CMR set 2	510	252	258
CMR set 3	508	249	259

**Table 3:** ED annotations

Total molecules	Positive	Negative	Uninformed	Similar positives	Similar No info
4634	199	3	4432	52	76

#### QSAR modelling

The datasets obtained as described above were used to build classifier models using conformal (Norinder et al. 2014; Svensson et al. 2018) random forest (Svetnik et al. 2003). The model building was carried out using the open-source modelling framework Flame (Pastor et al. 2020). In our analyses, we found that the model quality improvement obtained by RF hyperparameter optimization was not significant. Therefore, to obtain comparable results, we applied the settings shown in Table S1 to all the models. Since these models were conformal, the prediction consists of a couple of binary results indicating if the query compound belongs to class-0 (negative) or class-1 (positive). These are typically interpreted as positive when the compound belongs only to class-1, negative when it belongs only to class-0, and uncertain for compounds that belong to either both classes or neither.

The use of a conformal classifier allows considering prediction uncertainty (Svensson et al. 2018). At model building, the user is requested to introduce a significance level (e.g. 0.8). This value is used to assign the prediction to the classes mentioned above (positive, negative), guaranteeing that, as a maximum, 20% of misclassifications will be produced. The significance level can be varied depending on the situation where the model is to be applied, and the consequences of such changes are readily understandable (Norinder et al. 2014). For example, setting a high value of significance would reduce the uncertainty of the predictions, with the inconvenience that more predictions would be considered uncertain.

#### Ensemble models

In this study, we used ensemble models consisting of the combination of multiple qualitative models, using a methodology described previously (Pastor et al. 2020). To do so, we prepared several balanced datasets from the original CMR dataset, as described in the data preparation section. These were used to build individual classifiers using conformal RF. The ensemble model combines the results of these three models using a 'majority voting' approach. This means that query compounds were predicted using the three different models. The prediction results were then combined by counting the class-0 and class-1 predictions provided by the individual models. The final prediction is positive if the number of class-1 results is higher than the number of class-0, negative if the number of class-0 results is higher than the number of class-1, and uncertain if both numbers are similar.

After the models were created, these were applied to the non-informed compounds to obtain predicted values. It should be noted that thanks to the use of the conformal framework, every qualitative prediction is guaranteed to be correct with a certain degree of confidence (in this case, 80%) (Norinder et al. 2014).

#### Read across

When the balance of compounds in the positive and negative classes is not suitable for using classifiers, RAX was used as a “last resource” (Myatt et al. 2018; Patlewicz et al. 2017). For this study, we applied an analogue approach by characterizing the structural similarity between the query compounds (compounds with no annotation) and the closer annotated compounds in our database. The structural similarity was quantified using RDKit-computed fingerprints (Landrum 2006), using 2048 bits and the Tanimoto similarity metric (Bajusz et al. 2015). We set up a cutoff of 0.8: only compounds with higher structural similarity to an annotated analogue in the database than this cutoff are inferred to have similar toxicological properties. RAX was carried out using an ad hoc Python script (https://github.com/phi-grib/ED_RAX).

### Carcinogenesis

This assay was carried out by adapting the in vitro Bhas 42 cell transformation assay method described in the OECD Guidance Document No. 231 (2017) based on the work of Sasaki et al. (2014).

The Bhas 42 CTA has been developed for hazard identification of potential carcinogenicity of chemicals based on the measure of the morphological changes (transformation frequency) after exposure to a carcinogenic compound. It consists of two assays with a different test compound addition schedule to identify compounds with initiating activity (tumour initiators) or promoting activity (tumour promoters). Previously, a cell growth assay for 7 days is performed to set the assay doses of the CTA and another cell growth assay is concurrently performed to the CTA to verify that the selected doses meet the acceptance criteria for the tests. A compound was considered positive if it showed a statistically significant increase in the foci (morphologically altered cells forming discrete anchorage-independent colonies) frequency when compared with the vehicle-treated cells at least in two sequential concentrations of the five different concentrations evaluated.

### Endocrine disruption in androgen and oestrogen receptors

#### AR luciferase reporter assay

The transcriptional activation of androgen receptor (AR) assays was carried out by adapting the OECD protocol 458 (OECD 2020).

MDA-MB-453 cells (8000 cells/well) expressing an AR luciferase reporter were seeded into 384 white plates (Greiner 781098) in culture medium (DMEM (ATCC), 10% foetal bovine serum (Sigma Aldrich), 1% penicillin–streptomycin (Sigma-Aldrich), 75 µg/ml geneticin (Gibco) and incubated overnight at 37 °C 5% CO_2_ and 95% humidity. After this time, the cell culture medium was discarded and replaced by assay medium (DMEM (ATCC), 0.1% foetal bovine serum (Sigma-Aldrich), and 1% penicillin–streptomycin (Sigma Aldrich)).

For studying a putative agonist effect, compounds were added to the assay plate using an Echo 550 acoustic liquid handler and incubated overnight at 37 °C 5% CO_2_ and 95% humidity. For studying the putative antagonist effect, compounds were added using an Echo 550 acoustic liquid handler and incubated for 15 min at 37 °C 5% CO_2_ and 95% humidity. Then, 1 nM 5α-dihydrotestosterone (DHT) was added, and the cell plate was incubated overnight at 37 °C, 5% CO_2_, and 95% humidity. After compound incubation, the cell medium was removed, 5 µl of lysis buffer 1X (Promega) was added, and the cell plate was incubated with agitation 15 min at room temperature. A freeze–thaw cycle of 10 min at −80 °C and room temperature was performed. Then 25 µl of luciferase assay reagent (Promega) was added and luminescence was determined on an EnSpire plate reader (Perkin Elmer). Data was fitted to a four-parameter sigmoidal model with Prism 5.1 (Graphpad, Inc). A compound was labelled as an endocrine disruptor if in the agonist test its maximum effect related to the negative control was higher than 10% or if in the antagonist test the inhibition of the DHT effect was higher than 30%.

#### ER luciferase reporter assay

The transcriptional activation of oestrogen receptor (ER) assay was carried out by adapting the OECD protocol 455 (OECD 2021) and the method of Li et al. (2013).

ER luciferase reporter T47D cells (5000 cells/well) were seeded into 384 white plates (Greiner 781098) in culture medium (RPMI 1640 (Thermo Fisher Scientific 52400), 10% foetal bovine serum (Sigma Aldrich F9665), 1% penicillin–streptomycin (Sigma-Aldrich P0781), 75 µg/ml Geneticin (Gibco 10131)) and incubated overnight at 37 °C and 5% CO_2_. The following day, the cell culture medium was discarded and replaced by assay medium (RPMI 1640 (Thermo Fisher Scientific 52,400), 0.1% foetal bovine serum (Sigma Aldrich F9665), and 1% penicillin–streptomycin (Sigma Aldrich P0781).

The assay was performed as stated before for the AR assay using 17β-estradiol as agonist instead of DHT.

### Genotoxicity

#### Micronucleus test

In vitro cell micronucleus tests were carried out as described in the OECD protocol 487 (OECD 2016).

The in vitro micronucleus test is a genotoxicity test for the detection of micronuclei in the cytoplasm of interphase cells. Tests were conducted in the presence of the cytokinesis blocker cytochalasin B with three different treatment schedules: (i) short treatment for 6 h; (ii) short treatment for 6 h in the presence of an exogenous source of metabolic activation (cofactor supplemented S9 liver fraction); and (iii) extended treatment for 24 h.

CHO-K1 (Chinese hamster ovary cells, ATCC Item Number CCL-61) were cultured in DMEM F-12 containing 10% foetal bovine serum and 100 units/ml penicillin/100 μg/ml streptomycin (all from Sigma-Aldrich, St Louis, USA). Cells were seeded at 3000 cells/well in a collagen-coated 96-well plate in 100 μl. After 24 h, cells were incubated with CellTracker Orange CMTMR cellular dye solution (Invitrogen, Waltham, USA) for 1 h. Cells were then treated with compounds for 6 h, with or without S9 metabolic activation, or 24 h without S9 metabolic activation. In case of S9 metabolic activation, cofactor-supplemented Araclor 1254-induced male Sprague–Dawley rat liver S9 homogenate (BioIVT, Westbury, USA) was added at a final concentration of 0.1 mg/ml. After the 6-h compound treatment, cells were incubated with cytochalasin B (Alfa Aesar, Kandel, Germany) for 24 h. In case of 24-h treatment, cells were incubated with compounds in the presence of cytochalasin B and harvested after treatment. Benzo(a)pyrene, mitomycin C, and colchicine were used as positive controls for 6 h, with or without metabolic activation or 24 h treatments, respectively. After compound treatment and removal of the medium, cells were fixed with 3.7% formaldehyde (Merck, Darmstadt, Germany) and 5 µg/ml Hoechst 33,342 dye (Invitrogen, Waltham, USA) in BupH-modified DPBS saline packs (Thermo Scientific, Waltham, USA) for 20 min at room temperature. After several washes with BupH-modified DPBS, cells were analysed in an Operetta High-Content Imaging System (PerkinElmer, Waltham, USA). A compound was labelled as mutagen if at least one of the non-cytotoxic test concentrations exhibits a statistically significant increase compared with the concurrent negative control. Statistical significance was determined with the ANOVA test with significance level adjusted to *p* < 0.05.

### Acute toxicity

#### Zebrafish maintenance and toxicity determination

Adult zebrafish (*Danio rerio*, wild-type, strain AB) were maintained at 28ºC at a rate of 1 fish per litre of water, with a light–dark cycle of 14:10 h in the aquarium located at the veterinary facility (REGA code ES270280346401) of the University of Santiago de Compostela in Lugo, Spain. Zebrafish embryos were obtained mating adults based on previously described procedures (Westerfield 2000). Zebrafish care, use, and treatment were performed in agreement with the European Parliament and Council Directive 2010/63/EU on the protection of animals used for scientific purposes and the Spain Royal Decree 53/2013 on animal welfare standards. Experimental protocols were approved by the Ethical Committee of the University of Santiago de Compostela (15010/2015/001).

Acute toxicity was determined using the OECD236 fish embryo acute toxicity (FET) test (OECD guidelines). To assess the PBT potentiality of the analysed compounds, persistence and the logK_ow_ as a measure of bioaccumulation were extracted from public databases (https://comptox.epa.gov/dashboard, and https://echa.europa.eu/es/information-on-chemicals). To determine if a compound was PBT, the more conservative thresholds of the CLP for each property were considered and the three had to be positive for a given compound to be PBT.

### Reproductive toxicity

In REACH, most of the mammalian tests for chemical safety assessment are related to reproductive toxicity (ECHA 2020; Rovida and Hartung 2009; Sellick 2011) and consider complex molecular and cellular mechanisms determined in long-term experiments with very low throughput. To avoid the use of animals and to assay reproductive toxicity in a relevant 'non-animal' model adhering to the 3Rs rule, we used zebrafish embryos up to 120 hpf. Up to this developmental stage, the embryos are not considered animals according to European legislation (Sellick 2011). When they reach the stage of independent feeding, zebrafish embryos become subject to regulations for animal experimentation. We performed a set of previously described experiments to cover a wide range of potential reprotoxic effects, including terato-carcinogenesis, developmental neurotoxicity, and endocrine disruption.

Zebrafish embryos obtained and maintained as described previously for toxicity determination were used to determine (I) terato-carcinogenesis assayed as previously informed (Selderslaghs et al. 2009, 2012) with modifications. In brief, embryos were incubated for 0 hpf up to 120 hpf in the presence and absence of tested compounds. Each condition was assayed in triplicate with 12 embryos per condition per replicate. The appearance of selected characteristics and malformations with respect to normal development were quantified for the eyes, otoliths, heart, circulation, hatching, skeletal deformities, and body position. For each concentration tested, we assessed the mortality and the presence of malformations at 24, 48, 72, 96, and 120 hpf using a light microscope. When possible, the lethal effects (LC50) and terato-carcinogenic effects (EC50) were determined using the ToxRat software. Based on these values, a teratogenic index (TI) was calculated as LC50/EC50. Based on previous studies, we considered a compound to be a teratogen when TI > 2 at 120 hpf. When no TI could be calculated due to no malformations induction to a degree extensive enough to calculate the EC50 in the presence of high toxicity, the compound was considered a non-teratogen. (II) Neurotoxicity was assayed by evaluating the locomotor activity in embryos exposed to the tested compounds from 0 to 120 hpf using Zebrabox and Zebralab (ViewPoint). We tested three concentrations (24 embryos per concentration) below the NOEC to avoid readily toxic concentrations. After incubation with tested compounds for 120 hs in 96-well plates, the activity of larvae in response to transitions from dark to light was evaluated. The protocol involved an initial 10 min acclimation period inside the Zebrabox, followed by three cycles of light–dark of 10 min each. After the end of each cycle, the integrated activity of each embryo was registered. Statistical analysis was done using ANOVA, followed by the Tukey post hoc test. Differences were considered significant when *p* < 0.05. Dead embryos were not considered in the analysis. (III) The endocrine-disrupting effect in zebrafish embryos was assayed by real-time PCR as previously reported (Jarque et al. 2019) with modifications. In brief, 0 hpf embryos (12 embryos per condition in triplicate) were incubated with selected compounds for up to 120 hpf. After incubation, treated (embryos incubated at the concentration right below the NOEC) and control embryos were collected in RNA later and kept at − 20°C until use. RNA was extracted from the samples using the RNeasy Mini Kit (QIAGEN) and was followed by cDNA synthesis using random primers and the AffinityScript Multi Temperature cDNA Synthesis Kit (Agilent Technologies). Finally, real-time PCR was performed for eight target genes to obtain data on the estrogenic (cytochrome P450 family 19 subfamily A member 1: cyp19a1b, and vitellogenin: vtg1), androgenic (cytochrome P450 family 2 subfamily W member 1: cyp2k22, and cytosolic sulphotransferase 3: sult2st3), and thyroid hormone (thyroid peroxidase: tpo, thyroid hormone receptor alpha: tra, transthyretin: ttr, and iodothyronine deiodinase 2: dio2) effect of the tested compounds. Significant changes in gene expression between controls and treated samples were considered as a potential endocrine-disrupting effect.

A compound was considered as toxic for reproduction when positive for any of the three previously described experiments.

### Validation

To validate the accuracy of the predictive *in silico* models, we selected a collection of compounds representing different prediction results: positive and negative, more reliable, and less reliable. We aimed to obtain a representative prediction sample that can be compared prospectively with experimental results. The analysis of the agreement between both results can provide valuable information about the prediction quality and how the prediction quality depends on the value being predicted and the estimated prediction uncertainty.

The selection criteria used indicate that half of the selected compounds were predicted to be positive and half negative. Also, for these, half should have been predicted with a high degree of certainty and half with lower certainty. For selecting a series meeting these criteria, all the CII compounds with no experimental annotations were predicted by the *in silico* models, using conformal classifiers without defining the significance level. This yields for every prediction the probabilities that the compound belongs to class-0 (negative) or class-1 (positive). These two values can be used to quantify the significance of the prediction (as 1 minus the lowest of these *p* values) and the percentage of confidence (as the *p* value of the other class multiplied by 100) (Alvarsson et al. 2021).

After obtaining these values, the compounds are sorted based on the significance and the confidence. Then we select the top ten and the lowest ten because we want to stress test the reliability of our predictions and thus see if they reproduce the experimental results, even in unfavourable scenarios (lowest confidence predictions). Our expectations were that once ranked by their confidence, the experimental validation should be correct for the top ten, while for the lower ten we expect worse results. We did this selection twice, one for the positive predictions and another for the negative ones. The final result was a list with 20 positive and 20 negative compounds for each endpoint.

Finally, this list of candidate compounds was reduced by removing compounds difficult to obtain from commercial providers or with physicochemical properties that make them unsuitable for the experimental procedure. The final list of compounds that were tested experimentally can be seen in Table 4 (CMR), Table 5 (PBT), and Table 6 (ED).

**Table 4:** CMR selected substances

Name	CAS	Activity	*p* value 0	*p* value 1	Significance	Confidence (%)
Isethionic acid	107-36-8	0	0.750	0.003	0.997	75.0
Phenethyl benzoate	94-47-3	0	0.744	0.004	0.996	74.4
Phosphonic acid	1660-95-3	0	0.727	0.004	0.996	72.7
Reaction mass of cis-4-(isopropyl) cyclohexanemethanol and trans-4-(isopropyl) cyclohexanemethanol	5502-75-0	0	0.424	0.022	0.978	42.4
Sodium cocoyl glycinate	90387-74-9	0	0.397	0.023	0.978	39.7
*N*-[2-[(2-chloro-4,6-dinitrophenyl)azo]-5-(diethylamino)phenyl]acetamide	66557-45-7	0	0.355	0.022	0.978	35.5
2-Chloro-1-(4-chlorophenyl)propan-1-one	877-38-3	1	0.002	0.836	0.998	83.6
(R)-6-(isopropyl)-3-methylcyclohex-2-en-1-one	4573-50-6	1	0.002	0.800	0.998	80.0
2-Ethylhexylamine	104-75-6	1	0.002	0.792	0.998	79.2
4-Chloro-*N*-methylpyridine-2-carboxamide hydrochloride (1:1)	882167-77-3	1	0.013	0.560	0.987	56.0

**Table 5:** PBT selected substances

Name	CAS	Activity	*p* value0	*p* value1	Significance	Confidence (%)
2-Methylallyl alcohol	513-42-8	0	0.910	0.003	0.997	91.0
Polymethacrylic acid	25087-26-7	0	0.854	0.003	0.997	85.4
*N*-Isopropylacrylamide	2210-25-5	0	0.849	0.003	0.997	84.9
Nitrobenzene	98-95-3	0	0.813	0.003	0.997	81.3
Polyurethane	9009-54-5	0	0.709	0.003	0.997	70.9
2,2'-(Vinylenedi-p-phenylene)bisbenzoxazole	1533-45-5	0	0.216	0.033	0.968	21.6
2-Naphthalenecarboxamide, N-(5-chloro-2-methylphenyl)-3-hydroxy-	135-63-7	0	0.208	0.036	0.964	20.8
Methyl cinnamate	103-26-4	0	0.201	0.033	0.967	20.1
(1α,2α,3α,4β,5α,6β)-1,2,3,4,5,6-Hexachlorocyclohexane	319-86-8	1	0.007	0.845	0.993	84.5
1,2,3,5-Tetrachlorobenzene	634-90-2	1	0.013	0.718	0.987	71.8
1,2,3,4,5-Pentachlorobenzene	608-93-5	1	0.004	0.693	0.996	69.3
2,3,5-Trichlorophenol	933-78-8	1	0.023	0.244	0.977	24.4

**Table 6:** ED selected substances

Name	CAS	Activity	Similarity with a positive substance
2,5-Diaminotoluene sulphate	615–50-9	0	0.394
2,4-Xylidine	95–68-1	0	0.393
1,2,4-Trichloro-5-methylbenzene	23503–68-6	0	0.363
Citronellyl butyrate	141–16-2	0	0.356
2-Ethenylpyridine	100-69-6	0	0.282
Methyl dihydrojasmonate	24851-98-7	0	0.236
Cyclohexanol, 5-methyl-2-(1-methylethyl)-, (1S,2R,5S)-	15356-60-2	0	0.175
Propargyl bromide	106-96-7	0	0.080
1,2,3,5-Tetrachlorobenzene	634-90-2	1	0.906
Nonafluorovaleric acid	2706-90-3	1	0.877
2,3,5-Trichlorophenol	933-78-8	1	0.876
Phenanthrene	85-01-8	1	0.861

## Results

**Fig. 1:**
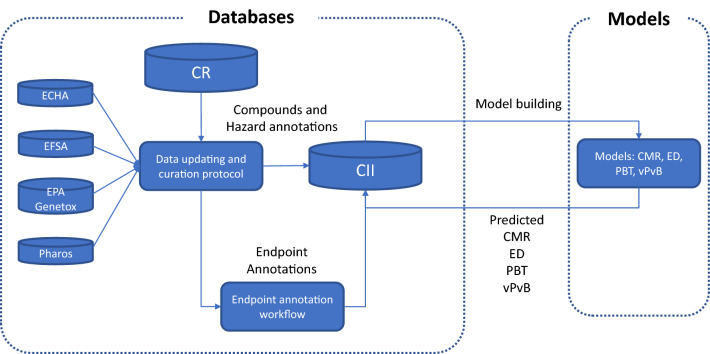
Schema of the system developed to compile toxicological information for substances used in LTI

In Fig. 1, we schematize the system we have developed to implement our strategy for the characterization of the chemical hazard of substances used in the LTI.

In the following sections, we describe the different components of this system and our efforts for validating the newly generated information.

### Databases

This project aims to generate a toxicological knowledge repository including the substances used in LTI. The number of these substances has been estimated in the order of 10,000 and about 3000 are commonly used (Roy Choudhury 2014), as mentioned in “Introduction”. To obtain a significant coverage of this chemical space, our database must incorporate at least this dimension.

Our project started in early 2019 by identifying substances used in LTI, as described in the Materials and methods section, and compiling available toxicological information for four selected endpoints (CMR, ED, PBT, and vPvB) from open access data sources. Compounds and toxicological information were stored in two databases: one specifically oriented to collect LTI compounds, annotated with experimental and predicted data, and another, more generic, used mainly to support the annotation assignment. A decision workflow was used to label every compound in the database as positive, negative, or No Information for the four considered endpoints using the annotations from the external sources, as described in the Materials and methods section. These databases were at the centre of a data collection, curation, and maintenance strategy involving other tools. In the Materials and methods section, we describe the details of the database development, update, and chemical structure curation.

In this period, new data sources were investigated, and some of them were included in the database updating protocol, resulting in a wider database with more annotations. This database updating protocol was run every three months, producing a new version of the database. The head version is then used in production, replacing previous versions which are stored only in compressed format.

At present (October 2020), the CII database contains 4634 substances with their hazards and endpoint annotations and regulations. The number of substances and annotations is detailed in Table 7. The endpoint annotations for CII are not completed for the vast majority of the entries. 4579 out of 4634 substances were not informed for one or more of the four considered endpoints. This is approximately 99% (98.81%). From a total of 18,536 possible annotations (4 endpoints for each compound), 15,661 are 'No Information'. Thus, around 84% (84.49%) of the annotations are missing.

**Table 7:** CII database in numbers

Compounds	4634
Curated compounds	4634
CAS	4673
EC	961
SMILES	4189
ECHA hazard Annotations	1531
Pharos hazard Annotations	563
Information sources	12
Compounds fully annotated	55
Compounds with at least one endpoint uninformed	4579
Total possible endpoint annotations	18,536
Distribution of endpoint annotations	No information: 15,661 Yes: 1642 No: 1194 Pending: 39

### Models

After collecting all available data from the selected data sources, many compounds still lack information for one or several of the considered endpoints. The next step was to fill existing knowledge gaps by using *in silico* prediction methods. In our strategy, the prediction results were considered provisional annotations, with an approximate value in the absence of experimental data. In the database, predicted annotations were kept separate from the experimental ones.

*In silico* prediction methods are NAM methodologies that use existing toxicological knowledge to infer the properties of the uninformed compounds. Details about how these methods were built are provided in the Materials and methods section. The first challenge we faced in this task was the extremely unbalanced distribution of positive and negative annotations for all the considered endpoints in the CII database. Unbalanced datasets are difficult to handle by most machine learning methods (Stewart 2020). Diverse approaches have been proposed to mitigate this issue. In this project, we compared two different algorithms to correct the class imbalance: simple oversampling (doubling the less populated class) and subsampling (data not shown). We decided to go on with the simple oversampling since it produces models of better predictive quality without losing information about the chemical space under study. For some endpoints (PBT and vPvB), the application of this method generates training series with a reasonable balance between positive and negative compounds (see Table 2). These were used to build classifier models of rather good quality, suitable for predicting the properties of uninformed compounds (see Table 8).

**Table 8:** Quality of PBT, vPvB, and CMR models

Endpoint	Sensitivity	Specificity	MCC^1^	Conformal coverage	Conformal accuracy
PBT	0.83	1	0.89	0.845	0.963
vPvB	0.862	1	0.907	0.864	0.965
CMR	0.981	0.989	0.97	0.873	0.985

^1^MCC: Matthews correlation coefficient

Unfortunately, this algorithm failed to produce adequately balanced datasets for the CMR endpoint. In this case, we generated three balanced datasets (see Materials and methods section) combined using a model ensemble of three classifiers. The sizes and positive/negative ratio of the three datasets are shown in Table 2 and the quality of the ensemble model in Table 8.

The best models obtained were used to predict uninformed compounds. The total amount of substances in the DB is 4634, but only 4293 have a structure. After removing problematic structures, we retained 3412. A total of 1222 substances, 881 with an untreatable structure and 341 without any structure, had to be processed using other methods, like a rule-based approach.

For making the prediction, 100 substances had to be discarded because they were not correctly processed by our modelling software (Flame). This led us to a total of 3312 substances.

Here, we summarize the results obtained when we applied these models for each of the considered endpoints. Further details can be found in the Materials and methods section.

#### CMR predictions

**Fig. 2:**
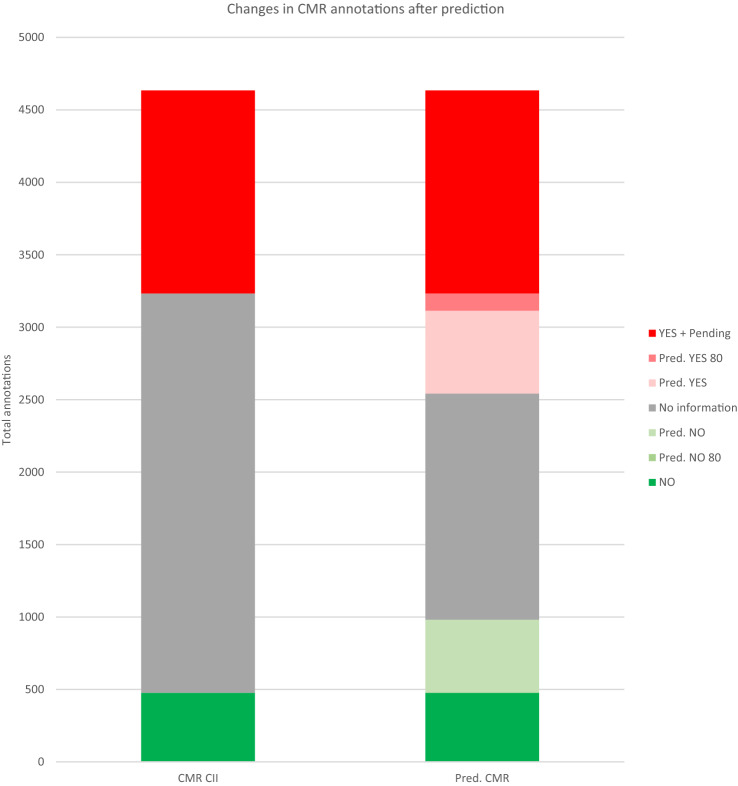
Changes in CMR annotations after prediction. In the left column, we have the annotations in CII. In the right column, we have the annotations in CII plus the predictions with and without the cutoff of 80% confidence. YES + Pending annotations increased from 1401 to 2092: 572 predictions under 80% of confidence and 119 higher than 80%. NO annotations increased from 477 to 982: 502 predictions under 80% of confidence and 3 (cannot be seen in the figure) above 80%. No Information annotations have decreased from 2756 to 1560 if all predictions are taken, but only to 2634 if the predictions higher than 80% are considered. The amount of non-informed compounds filled is 43.4%, but for high-confidence predictions only 4.43%

The prediction results are shown in Table 9. From the 3312 predictions obtained, 1423 were positive, 813 negative and 1076 uncertain. The conformal coverage (non-uncertain compounds) of this prediction was of 67.5%. Regarding the substances already annotated in CII, this exercise allows to categorize 1196 non-informed substances, from which 691 (57.8%) were positive and 505 (42.2%) negative. If we focus our attention only on high-confidence predictions (confidence equal or higher to 80%), the model will identify only 122 new annotations (3.7% of the original 3312 predictions), 119 positives and 3 negatives. After the prediction, the total number of positive substances increased from 1401 to 2092 and the total number of negatives from 477 to 982. If only the high-confidence (over 80%) predictions are considered, then the new number of positives is 1520 and 480 for negatives, as can be seen in Fig. 2.

**Table 9:** CMR prediction dataset results

Endpoint	Model type	Total molecules	Positive	Uncertain	Negative	Positive, not present in CII	Negative, not present in CII	Positive, not in CII and > = 80% confidence	Negative, not in CII and > = 80% confidence
CMR	Conformal ensemble	3312	1423	1076	813	691	505	119	3

#### PBT–vPvB

**Fig. 3:**
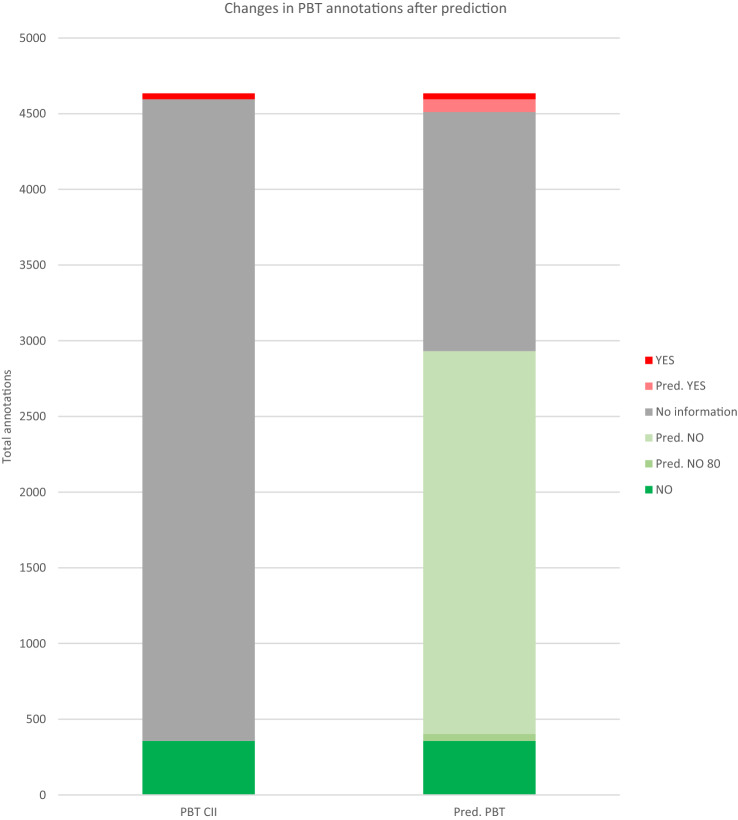
Changes in PBT annotations after prediction. In the left column we have the annotations in CII. In the right column we have the annotations in CII plus the predictions with and without the cutoff of 80% confidence. YES annotations increased from 38 to 123: 85 predictions under 80% of confidence, but none higher than 80%. NO annotations increased from 357 to 2932: 2528 predictions under 80% of confidence and 47 above 80%. No information annotations decreased from 4239 to 1579 if all predictions are taken, but only to 4192 if the predictions higher than 80% are considered. The amount of non-informed compounds filled is 62.75%, but for high-confidence predictions only 1.11%

**Fig. 4:**
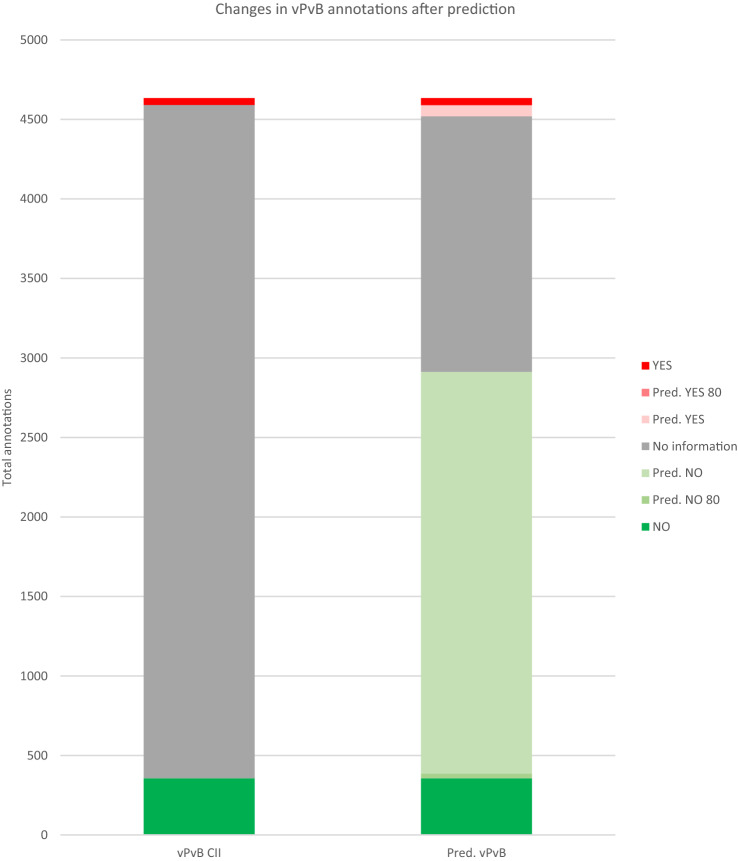
Changes in vPvB annotations after prediction. In the left column, we have the annotations in CII. In the right column, we have the annotations in CII plus the predictions with and without the cutoff of 80% confidence. YES annotations increased from 43 to 115: 69 predictions under 80% of confidence and 3 (cannot be seen here) higher than 80%. NO annotations increased from 357 to 2912: 2.526 predictions under 80% of confidence and 29 above 80%. No Information annotations decreased from 4234 to 1607 if all predictions are taken, but only to 4202 if the predictions higher than 80% are considered. The amount of non-informed compounds filled is 62.04%, but for high-confidence predictions only 0.75%

Again, the total number of possible predictions is 3312. The PBT model produced predictions for 2920 molecules (coverage of 88%), and the vPvB predicted 2888 molecules (coverage of 87%). But again, if we consider only the predictions with a confidence of 80% or more, the coverage is rather small (1.42%). In the PBT case, the positive predictions go from 85 to 0 and the negative ones from 2575 to 47. Regarding vPvB, the coverage is of only 0.97%, positive predictions decrease from 72 to 3, and the negative ones from 2555 to 29. These results are shown in Table 10 and Figs. 3 and 4.

**Table 10:** PBT and vPvB prediction datasets results

Endpoint	Imbalance correction	Total molecules	Positive	Uncertain	Negative	Positive, not present in CII	Negative, not present in CII	Positive, not in CII and > = 80% confidence	Negative, not in CII and > = 80% confidence
PBT	Simple oversampling	3312	121	392	2799	85	2575	0	47
vPvB	Simple oversampling	3312	110	424	2778	72	2555	3	29

#### Endocrine disruptor

The ED endpoint represents an extreme situation where the training series contains only 3 negatives and 199 positive substances (see Table 3). In this case, the lack of negative annotations forced us to use RAX, as described in the Materials and methods section. We computed structural similarity (using RDKit fingerprints and Tanimoto similarity index) between all the positive molecules and all the No Information, setting up a similarity cutoff of 0.8.

**Fig. 5:**
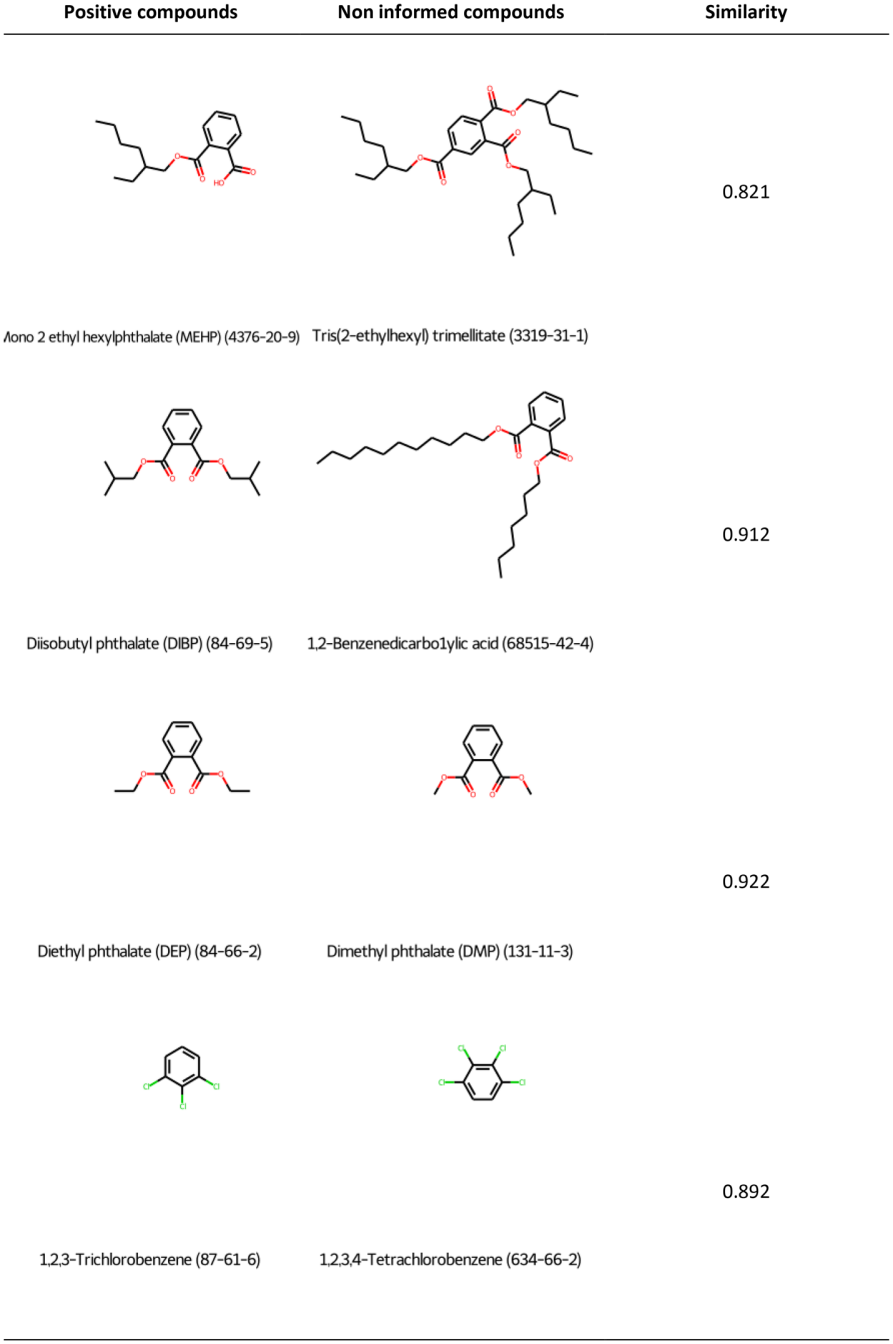
An example of the RAX result for the ED endpoint. In the left column, a set of structures of positive compounds in CII is shown, and in the middle column, a set of non-informed compounds in CII that are very similar to the positive ones is depicted. In the right column, we can see the value of the similarity between each pair of compounds (Tanimoto index, computed using RDKit fingerprints, as described in the Materials and methods section)

This analysis found that 76 non-informed compounds were very similar to a positive substance. It should be noted that this is a conservative criterion in the sense that even compounds with a mild structural similarity with a positive compound were considered as positive. We have included in Fig. 5 an example of the compounds assigned with a positive annotation to illustrate this aspect.

### Experimental validation

Before the predicted annotations could be considered for practical applications, it is necessary to validate their reliability to understand better how often these predictions reproduce the experimental results. With this aim, we designed a prospective validation study, where we selected a validation series containing a balanced set of compounds predicted as positive and as negative for each of our predictive models (CMR, PBT, vPvB, and ED). Also, we made sure that these series include both highly reliable and less reliable predictions, as estimated by the conformal classifiers (see Materials and methods section for further details).

The compounds were selected as explained in the Method section. The substances were acquired from commercial providers and evaluated experimentally using the best methods available in our team for reproducing the predicted annotations. Some compounds were not tested due to availability issues or physicochemical properties.

Before discussing the results of this validation exercise, the experimental methods are summarized here (see the Materials and methods section for a more detailed description). No suitable experimental method was available for the vPvB endpoint, and therefore the experimental validation of our vPvB predictions is pending. Also, it should be noted that the in vitro methods are only an approximation to the endpoint evaluation, and some of them do not cover all possible mechanisms. For example, for ED validation we selected in vitro methods for detecting interactions with the androgen and oestrogen receptors, the main receptors involved in reproduction, but there are other molecular mechanisms that can lead to endocrine disruption in vivo if they are altered by a xenobiotic compound. This fact should be considered in the interpretation of the results, in the sense that a positive in our experimental assays can be interpreted as a positive annotation, but a negative result should not be interpreted as a negative annotation since the compound can still be positive due to a mechanism not well covered by our test. In other words, our battery of tests can be considered specific, but not too sensible.

#### CMR assays

The carcinogenesis potential of the compound was assayed by means of measuring the frequency of transformed foci (morphologically altered cells that form discrete anchorage-independent colonies) in the Bhas 42 cell line, which can be extrapolated to an assessment of carcinogenic potential. In the transformation assay, the incubation of compounds with two different treatment schedules allowed to identify the initiating or promoting carcinogenic potential of the compounds. Three of the tested compounds showed a statistically significant increase in the foci frequency when compared to the vehicle-treated cells at least in two sequential concentrations of the five different concentrations evaluated and were considered potential carcinogenic compounds according to the OECD guideline. The concentrations were selected on the basis of a dose-finding assay measuring compound cytotoxicity as suggested by Sasaki et al (2014, 2015).

None of the tested compounds showed a significant increase in micronuclei formation at any of the three concentrations evaluated at the different treatment schedules (6 h with or without metabolic activation or 24 h without metabolic activation). Therefore, the CMR safety warning of the compounds was based on the carcinogenic and reprotoxic effects only.

Reprotoxicity was assessed using three methodologies, teratogenic effect, neurotoxicity, and endocrine-disrupting effect, as pointed out in “Materials and methods”. A compound was considered reprotoxic if it was proved positive in any of the assays. None of the compounds analysed showed terato-carcinogenic effect. (R)-6-(isopropyl)-3-methylcyclohex-2-en-1-one was neurotoxic reducing up to 80% the embryo integrated activity at a concentration of 30 mg/L Based on this result, this compound was reported to be reprotoxic. However, the neurotoxic effect was observed at concentrations > 1 mM which are not physiologically relevant. Actually, lower concentrations (1mg/L) showed no neurotoxicity. 2-Ethylhexylamine and N-[2-[(2-chloro-4,6-dinitrophenyl)azo]-5-(diethylamino)phenyl]acetamide induced the cytochrome P450 2 K and the first also induced vitellogenin. These two compounds were the only positives for endocrine disruption and were reported to be reprotoxic.

#### ED assays of androgen and oestrogen receptors

ED was assessed by transactivation in vitro assays to identify agonists and antagonists of the androgen and oestrogen receptors. All the compounds detected as endocrine disruptors behaved as antagonists of androgen receptors with percentages of inhibition of androgen receptors higher than 30% as indicated by the OCDE. The IC50 values obtained were in the range between 0.29 and 470 μM. None of the compounds behaved as agonists of AR or ER receptors or as ER antagonists.

#### Ecotoxicity

Ecotoxicity of selected compounds was screened with the FET assay to determine acute toxicity and using available predictions of persistence and bioaccumulation. Based on these data, pentachlorobenzene and 1,2,3,5-tetrachlorobenzene were identified as potential PBT compounds. The compound δ-HCH was highly toxic and persistent. While bioaccumulation prediction by logK_ow_ (3,95) leaves this compound out of the PBT category, experimental determination of the BCF would be appropriate for this compound since it was ruled out as PBT due to a logK_ow_ of 3.95 which is quite near the 4,5 cut off value established by REACH. According to this same regulation, a bioaccumulation factor higher than 2000 would confirm this compound to be PBT. We conducted the bioaccumulation experiments to confirm if this compound could be classified as PBT according to REACH.

### Validation results and discussion

Before entering into details, we can state that the validation results confirm that all of our predictive models are performing as expected. When the predictions were labelled as having high confidence, the prediction results were in agreement with the experimental results in all instances. For the rest of the predictions, a few disagreements between the experimental results and predictions were observed.

These results confirm the validity of our proposed protocol: it is possible to use *in silico* prediction methods for gap filling, and the quality of the predictions is in good agreement with experimental methods, particularly in situations when we have good quality data. On the other hand, the results also confirm the expected limitations of the modelling approach. The good news is that the reliability metrics provided by the models were able to identify accurately the compounds more likely to be incorrectly assessed for the considered endpoints.

### RAX: ED

As explained before, the use of RAX was forced by the lack of suitable data and cannot be expected to produce reliable predictions. As a maximum, it will indicate the presence of compounds highly similar to positive ones that will be assigned a positive value. We selected a set of compounds that included four positive annotated compounds and eight non-informed compounds that were not similar to any positive compounds. Also, the experimental group analysed the endocrine-disrupting activity for the CMR compounds. As it can be seen in Table 11, the positive compounds assignment was in agreement with experimental results for two of them (phenantrene and 2,3,5-triclorophenol) and in disagreement for the other two (nonafluorovaleric acid and 1,2,3,5-tetrachlorobenzene). Before considering these two last results as a false positive, we must note that the experimental methods used to assess ED were not comprehensive and focused only on AR and ER receptors. The endocrine-disrupting activity of these two compounds is due to thyroid system affection as reported (Coperchini et al. 2021; Croce et al. 2019) for nonafluorovaleric acid and (Chu et al. 1983; den Besten et al. 1991) for 1,2,3,5-tetrachlorobenzene. Therefore, in this case, the disagreement detected is more a consequence of our experimental models' limitations, and the *in silico* methods have produced a more informative result.

**Table 11:** ED experimental result comparison with RAX

Name	CAS-RN	Experimental result	RAX result	Similarity to closer positive
Nonafluorovaleric acid	2706–90-3	No	Yes	0.877
1,2,3,5-Tetrachlorobenzene	634–90-2	No	Yes	0.906
Phenanthrene	85–01-8	Yes	Yes	0.861
2,3,5-Trichlorophenol	933–78-8	Yes	Yes	0.876
2-Ethenylpyridine	100–69-6	Yes	–	0.282
Propargyl bromide	106–96-7	Yes	–	0.080
Citronellyl butyrate	141–16-2	Not analysed	–	0.356
Cyclohexanol, 5-methyl-2-(1-methylethyl)-, (1S,2R,5S)-	15356–60-2	No	–	0.175
1,2,4-Trichloro-5-methylbenzene	23503–68-6	Not analysed	–	0.363
Methyl dihydrojasmonate	24851–98-7	Yes	–	0.236
2,5-Diaminotoluene sulphate	615–50-9	No	–	0.394
2-Ethylhexylamine	104–75-6	Yes	–	0.455
Phosphonic acid	1660–95-3	No	–	0.452
(R)-6-(isopropyl)-3-methylcyclohex-2-en-1-one	4573–50-6	No	–	0.500
Reaction mass of cis-4-(isopropyl) cyclohexanemethanol and trans-4-(isopropyl) cyclohexanemethanol	5502–75-0	No	–	0.484
*N*-[2-[(2-chloro-4,6-dinitrophenyl)azo]-5-(diethylamino)phenyl]acetamide	66557–45-7	Yes	–	0.524
4-Chloro-*N*-methylpyridine-2-carboxamide hydrochloride (1:1)	882167–77-3	No	–	0.520
Sodium cocoyl glycinate	90387–74-9	No	–	0.474
Phenethyl benzoate	94–47-3	No	–	0.643

It is also interesting to highlight that the experimental method detected a number of ED-positive compounds that the RAX was not able to assess due to the low similarity with annotated compounds in CII. This fact further highlights the importance of including as many experimental annotations as possible in CII. This is also a cautionary result for avoiding interpreting "non-assessment" results obtained from RAX as negatives, something that can clearly lead to wrong conclusions.

### QSAR: CMR

For comparison with the prediction results, the results of the carcinogenicity, micronucleus, and reprotoxicity assays were combined using a logical OR operator. Therefore, we consider that compounds with a positive result for any of these three experimental assays is a CMR positive. These results are shown in Table 12.

**Table 12:** CMR experimental results comparison with QSAR model

Name	CAS	Carcinogen (C)	Micronucleus assay (M)	Reprotoxicity (R)					*In silico*	Confidence in prediction	CII
				Experimental conclusion	Teratogen	Neurotoxic		Endocrine disruptor			
2-Ethylhexylamine	104-75-6	Yes	Negative	Yes	No		No	Yes	Yes	High	No info
Phosphonic acid	1660-95-3	No	Negative	No	No		No	No	No	High	No info
(R)-6-(isopropyl)-3-methylcyclohex-2-en-1-one	4573-50-6	No	Negative	Yes	No		Yes^a^	No	Yes	High	No info
Reaction mass of cis-4-(isopropyl) cyclohexanemethanol and trans-4-(isopropyl) cyclohexanemethanol	5502-75-0	Yes	Negative	No	No		No	No	No	Low	No info
*N*-[2-[(2-chloro-4,6-dinitrophenyl)azo]-5-(diethylamino)phenyl]acetamide	66557-45-7	Yes	Negative	Yes	No		No	Yes	No	Low	No info
4-Chloro-*N*-methylpyridine-2-carboxamide hydrochloride (1:1)	882167-77-3	No	Negative	No	No		No	No	Yes	Low	No info
Sodium cocoyl glycinate	90387-74-9	No	Negative	No	No		No	No	No	Low	No info
Phenethyl benzoate	94-47-3	No	Negative	No	No		No	No	No	High	No info
Isethionic acid	107-36-8	–	Negative	–	–		–	–	NO	High	No info
2-Chloro-1-(4-chlorophenyl)propan-1-one	877-38-3	–	Negative	–	–		–	–	YES	High	No info

^a^While neurotoxic, the effective concentration ~ 1.9 mM is far from being metabolically relevant. Lower concentrations (1 mg/L) showed no neurotoxicity

When the experimental results combined in this way were compared with highly reliable predictions, there is a nearly complete coincidence. The only compound for which the experimental results did not confirm the prediction is 2-chloro-1-(4-chlorophenyl) propan-1-one (877–38-3). This compound was predicted to be positive and the experimental results for micronucleus assay were negative, but the reprotoxicity assay could not be completed due to technical problems (the compound is too volatile). Therefore, we can only conclude that our prediction cannot be confirmed in this case due to the absence of complete experimental results.

Conversely, lower reliable predictions show more discrepancies: 5502-75-0 and 66557-45-7 have a NO in the prediction and a YES in the experimental result, whereas 882167-77-3 has a YES in the prediction and NO in the experimental result. These discrepancies were expected since the validation series contained representatives of predictions obtained with different degrees of quality precisely for this purpose and indeed indicate that our prediction reliability indexes are able to assess the degree of accuracy of the prediction results.

### QSAR: PBT

For this endpoint, the results of the persistence and bioaccumulation assays were combined using a logical AND operator, since the PBT labelling assigns a positive value only when the compounds are identified to be persistent, bioaccumulative, and toxic (REACH, 1.1., PBT Substances 2021).

The comparison of the experimental annotation with the high-confidence predictions (Table 13) shows a single discrepancy: *δ*-HCH (319-86-8) was predicted as positive, while the experimental result is negative. However, when investigating more closely these experimental results, we see that this compound has a *K*_ow_ of 3.95, which is very close to the REACH value of 4.5 to be considered as PBT. Thus, δ-HCH is in a twilight zone close to being a PBT. Even this, the BCF higher than 2000 would rule out that this compound is PBT according to REACH. We are conducting experiments to calculate the BCF.

**Table 13:** PBT experimental results comparison with QSAR model

Name	CAS	Persistence (water)	Bioaccumulation	Potential PBT	*In silico*	Confidence in prediction	CII
Pentachlorobenzene	608–93-5	Yes	Yes (5.17^e^)	YES	YES	High	No info
1,2,3,5-Tetrachlorobenzene	634–90-2	Yes	Yes (4.61^e^)	YES	YES	High	No info
2-Methyl-2-propen-1-ol	513–42-8	No	No (0.60^p^)	NO	NO	High	No info
2,3,5-Trichlorophenol	933–78-8	No	No (3.84^e^)	NO	YES	Low	No info
Methacrylic acid	79–41-4	No	No (0.93^e^)	NO	–		No info
Methyl cinnamate	103–26-4	No	No (2.62^e^)	NO	NO	Low	No info
*N*-Ethylurea	625–52-5	No	No (-0.74^e^)	NO	–		No info
*N*-Isopropylacrylamide	2210–25-5	Yes	No (0.41^p^)	NO	NO	High	No info
δ-HCH	319–86-8	Yes	No (3.95^e^)	NO	YES	High	No info
Nitrobenzene	98–95-3	No	No (1.85^e^)	NO	NO	High	No info

^e^Experimental value

^p^Predicted value

In the case of the lower confidence predictions, there is only one discrepancy as well: 2,3,5-trichlorophenol (933–78-8) has been annotated as positive, while the experiments suggest that this is not a potential PBT compound.

## Conclusions

Here, we presented a long-term strategy to inform the toxicological properties of a significant portion of the compounds used in the LTI. The strategy combines the systematic collection of information from open access data sources, applying logical decision workflows to integrate all the annotations into simple labelling, and using *in silico* prediction methods for gap filling. Due to resource limitations, the method has been focused on a few endpoints: CMR, ED, PBT, and vPvB.

The substances used in LTI are very diverse and cover a region of the chemical space that has rarely been explored. Working with such a diverse group of substances is complex and time-consuming. We had to implement methods to handle complex substances with no associated chemical structure and to classify untreatable structures in diverse categories. We consider that the tools developed in our group for collecting this data, curating it, and the step-by-step protocols to analyse are worth reporting and sharing.

The method described is currently running, producing updated versions of the database every 3 months, enriching it with new compounds and annotation, and integrating more and improved predictions. Originally, the database contained 4634 compounds and 2875 endpoint annotations (YES: 1642, NO: 1194, Pending: 39). With our predictions, we increased these annotations to 9156 (YES: 2330, NO: 6826). These new predicted annotations will be included in the following versions of the database. Still, despite our efforts, there are a large number of annotations missing. Considering the total possible number of annotations (4634 × 4 = 18,536), the 9156 reported here are 49.39% of all annotations. When only high-confidence predictions are taken into account, the increase is 3037 (YES: 1764, NO: 1273), which are the 16.4% of the total possible annotations. This means that 50.61% of these annotations, (83.6% if only high-confidence predictions are used) are still unknown, which give us the idea of how much work is ahead if we want to complete an exhaustive safety characterization of all the compounds used in the LTI. Such investment is indeed justified by the preliminary results presented here, since a significant proportion of the predictions generated for non-informed compounds were indeed positive. These results further justify the ongoing efforts of the European Commission, as described in its Chemicals Strategy for Sustainability (Conto 2021) to progress in the hazards and risk assessment of chemicals used by the industry.

Part of the present work was devoted to reporting the results of a prospective validation exercise of the *in silico* methods used to fill the gaps in our database. The validation was rather strict and studied a series of compounds, including both reliable and unreliable predictions. The results obtained show that these methods can be a useful tool for producing provisional annotations, particularly if the predictions' reliability is taken into consideration. Another outcome of this exercise is a clearer idea of the prediction accuracy that can be expected in different situations, emphasizing the importance of keeping collecting high-quality data, as this is the main limiting factor of the predictive quality of any *in silico* method.

## Supplementary Information

Below is the link to the electronic supplementary material.

Supplementary file1 (DOCX 2710 KB)

Supplementary file2 (PPTX 45 KB)

Supplementary file3 (PPTX 49 KB)

Supplementary file4 (PPTX 47 KB)

Supplementary file5 (PPTX 47 KB)
