# Supplementary material for: A continuous in silico learning strategy to identify safety liabilities in compounds used in the leather and textile industry
Source: Arch Toxicol. 2023 Feb 12;97(4):1091–111. doi: 10.1007/s00204-023-03459-7 (PMC10025185; doi:10.1007/s00204-023-03459-7)

**Supplementary material**

| **Table S1** Flame parameters | |
| --- | --- |
| Parameter | Selected option |
| Input type | Molecule / Ensemble model |
| Data type | Qualitative |
| Normalize method | ChEMBL |
| Model autoscaling | StandardScaler |
| Molecular descriptors | RDKit_properties, RDKit_md |
| Learner | Random Forest (RF), conformal, confidence 0.8 |
| Model validation | k-fold 5 |
| feature selection | None |
| imbalance | None |
| RF parameters | - Class_weight: balanced - Max_depth: max_depth - Max_features: sqrt - N_estimators: 200 - Out-of-bag score (oob_score): active - Random_state: 46 |


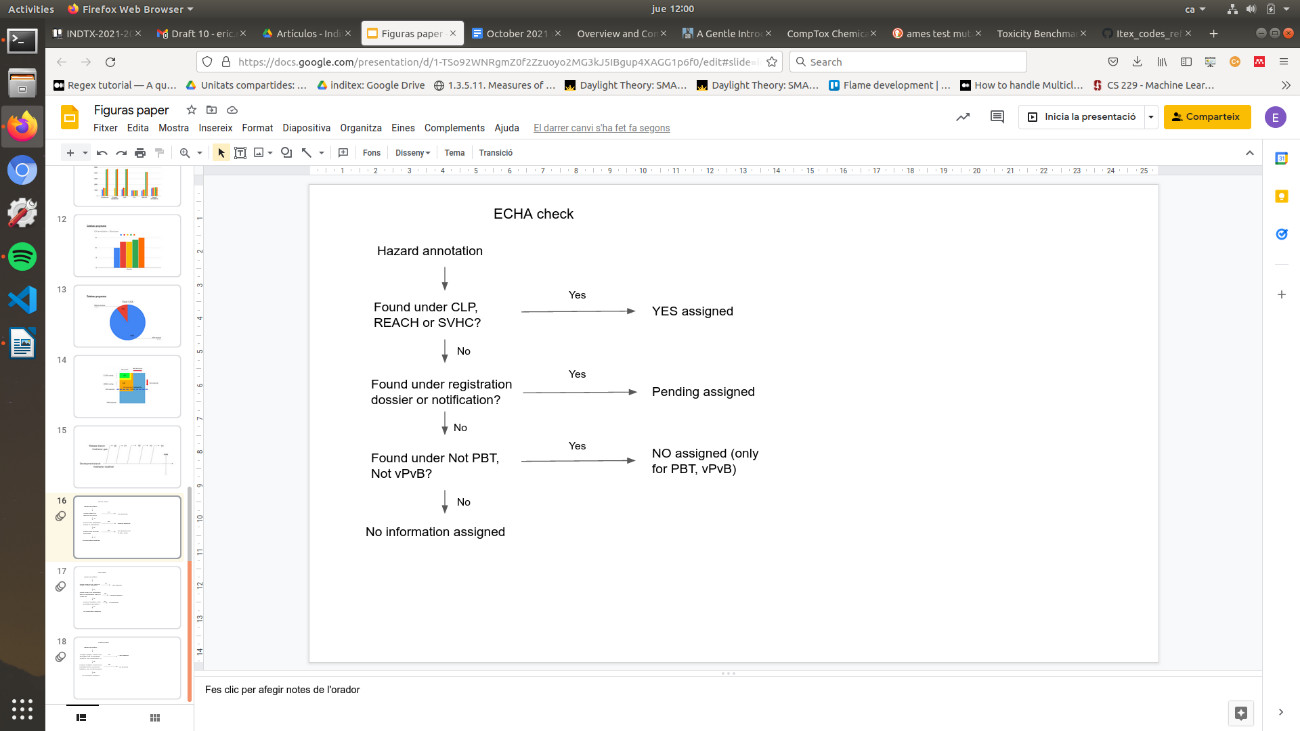

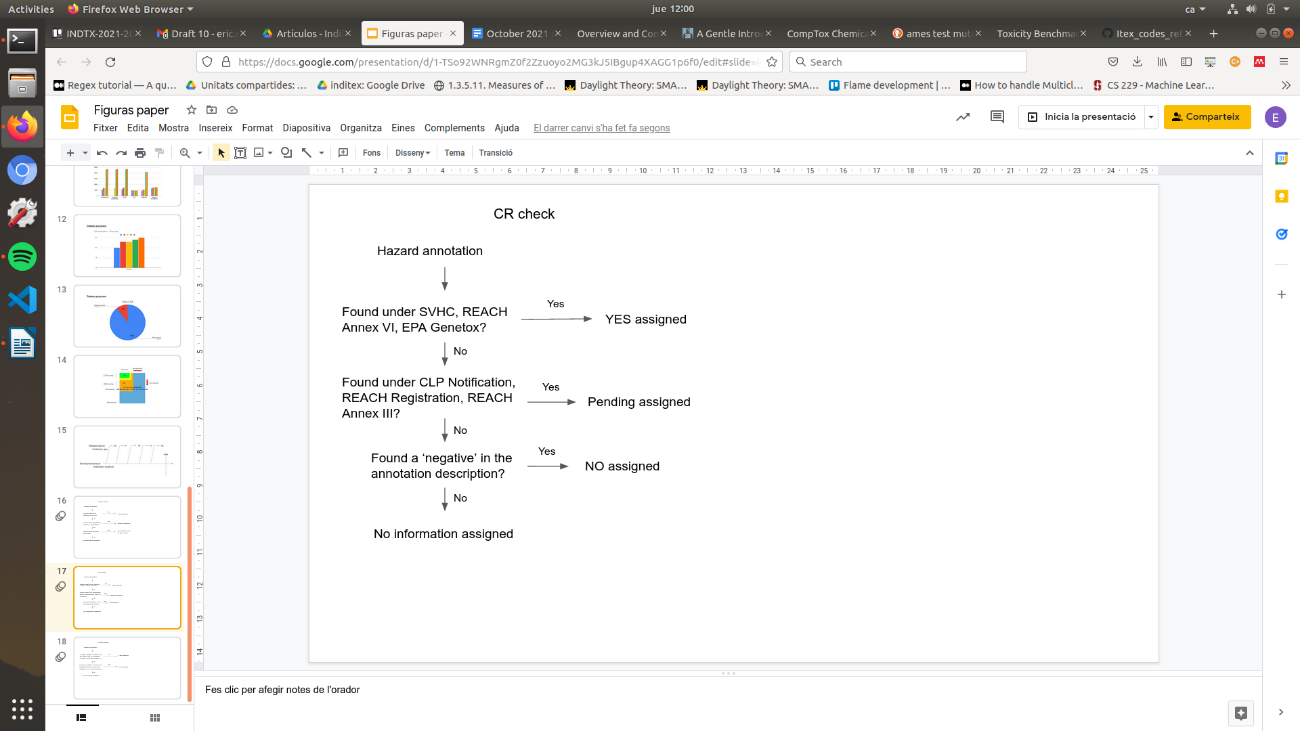

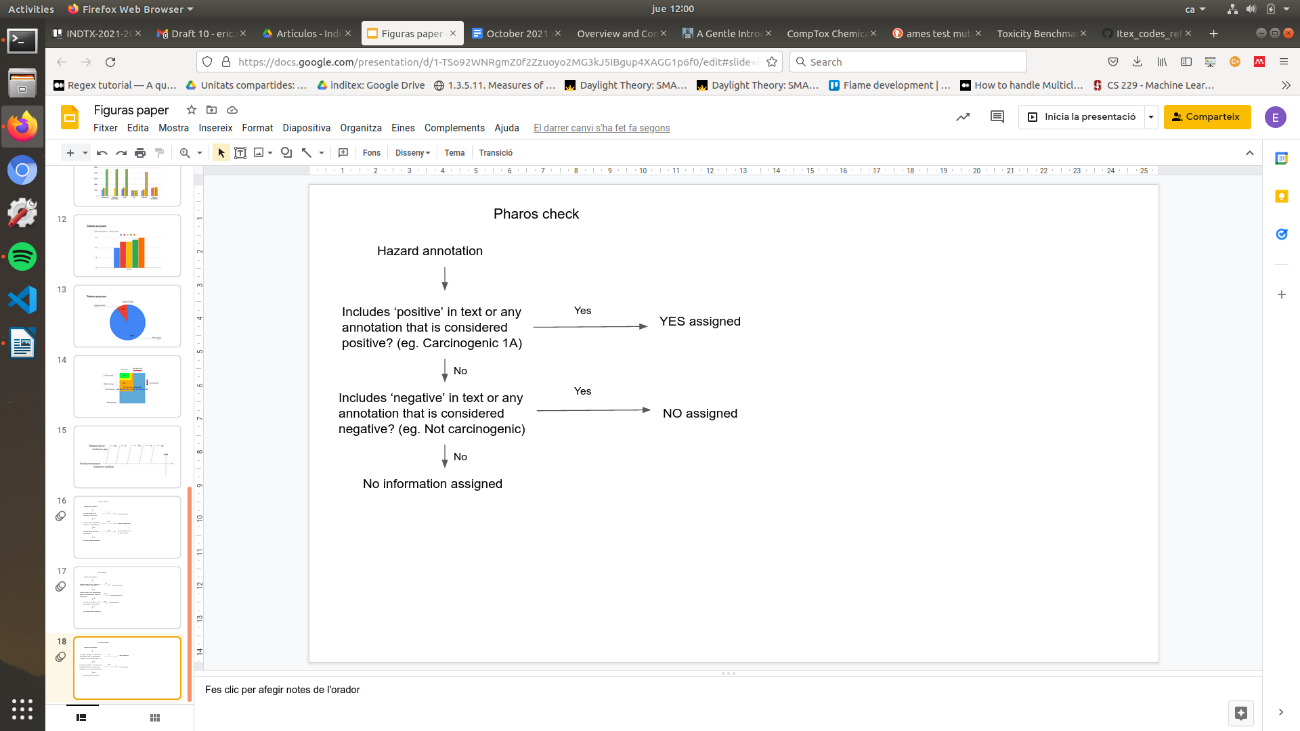


**Fig. S1** The three steps of the workflow used to assign endpoint annotations are depicted. The order is 1st ECHA check, 2nd CR check and 3d Pharos check

**Fig. S2** CMR prediction results. Out of the 1423 substances predicted as positive, we removed the duplicates and we got 1412. There are 1401 CMR positive (YES + Pending) substances in CII, which turn out to be 1048 after removing problematic substances and duplicates. When comparing the predicted results with the substances in our database, we saw that out of the 1048 positive substances in CII, only 721 were predicted as positive while 327 were not. Instead, 266 were predicted as uncertain, 50 as negative and 11 were removed before creating the models. That left us with 691 substances predicted as positive that were annotated as No information. Finally, when we remove the substances with less than 80% of confidence in the prediction, we end up having 119 positive substances. When it comes to negative predictions, we predicted 813. After duplicate removal we remained with 802. In CII there’s a total of 477 negative CMR substances, which after removing problematic structures and duplicates it becomes 330. From those 330, 297 were predicted as negative and 33 were not. Those 33 included 25 uncertain, 3 positive and 5 were removed before creating the models. That means that we have predicted 505 substances as negative that were annotated as No Information. After removing the substances with less than 80% of confidence in the prediction, we have left only 3 negative substances.


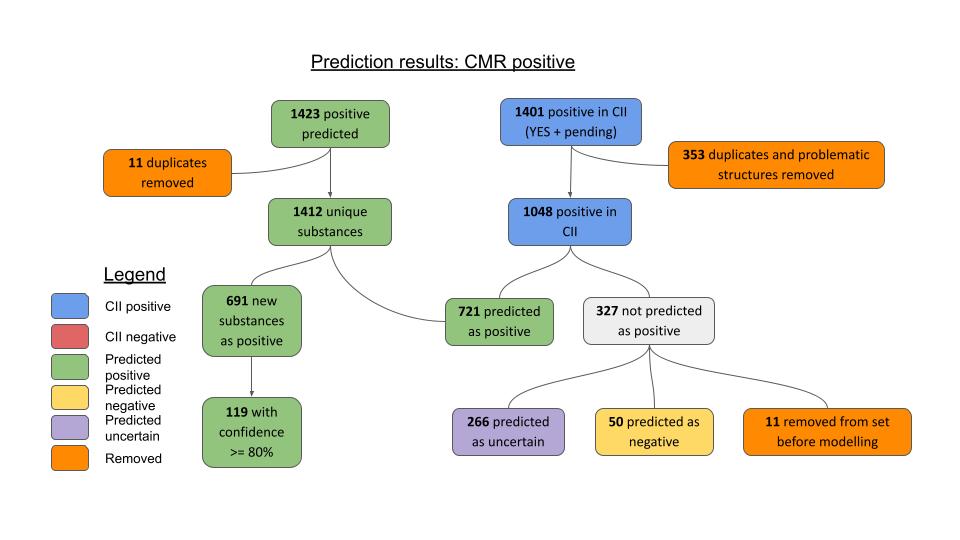

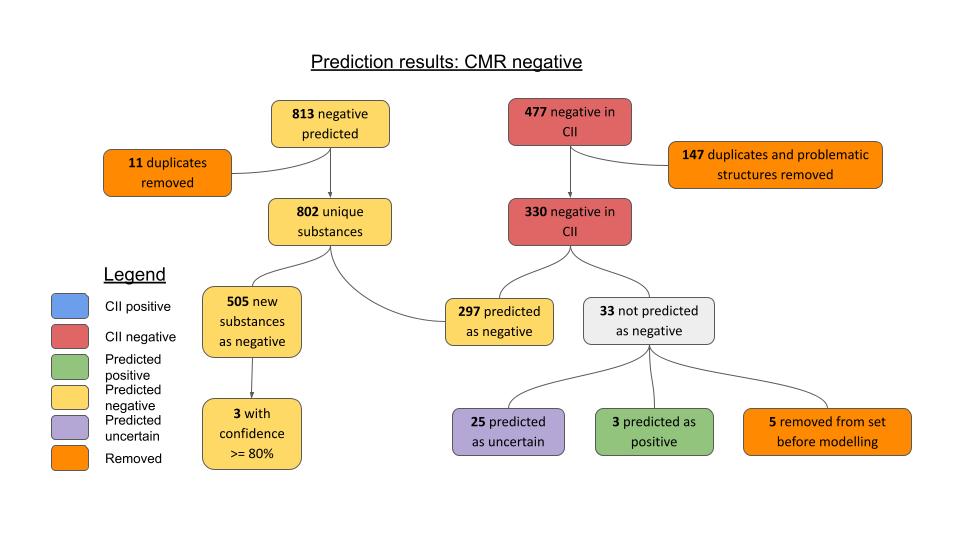


**Fig. S3** PBT prediction results. We predicted 121 positive substances, and after removing duplicates we remained with 112. In CII there are 38 positive substances. After the removal of problematic structures and duplicates we got 29. From these 29, 27 were predicted as positive by our model, leaving 2 out that were predicted as uncertain. That means that we predicted 85 new compounds as positive that were annotated as No information. From these substances, when we removed the ones that had predictions with less confidence than 80%, we ended up having 0 positives. Regarding the negative predictions, we obtained 2799. After duplicate removal we got 2774. There are 357 negative substances in CII but with a valid structure and no duplications the number is reduced to 205. From these 205, 199 were predicted as negative and when we removed the ones with less than 80% of confidence in the prediction we obtained 47 substances. Also, there were 4 predicted as uncertain and 2 were not processed by Flame. We can say that we have predicted a negative annotation for 2575 new substances and only 47 were with high confidence.


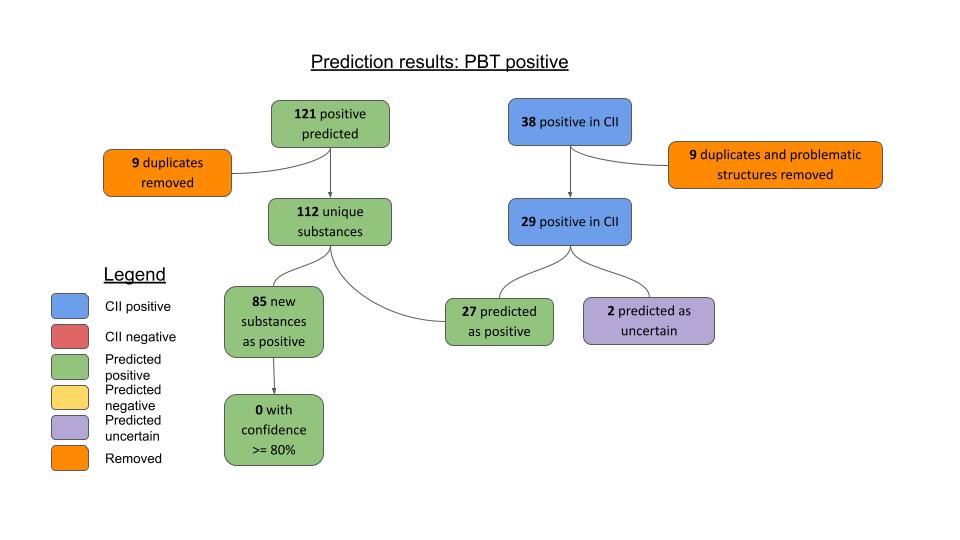

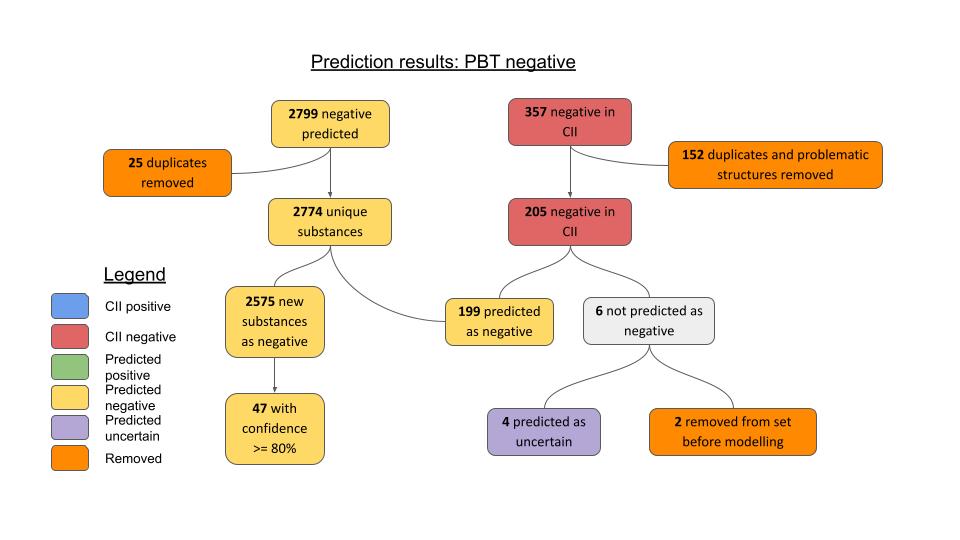


**Fig. S4** vPvB prediction results. We predicted 110 positive substances, which after removing duplicates we retained 102. There are 43 positives in CII. After removing problematic structures and duplicates they turn into 34. From these 34, 30 were predicted as positive and 4 as uncertain. We can say that we have predicted 72 new compounds as positive for vPvB. When we checked for the ones with high confidence in the prediction we ended up having 3 positive substances. For the negative ones, from the 2778 predicted we got 2753 after removing duplicates. There are 357 negatives in CII and without duplicates and problematic structures there are 205. From these 205, 198 were predicted as negative, 5 as uncertain and 2 were not processed by Flame. We can conclude that we have predicted a negative annotation for 2555 new substances. When we checked for the ones with high confidence in the prediction we ended up having 29 negative substances.


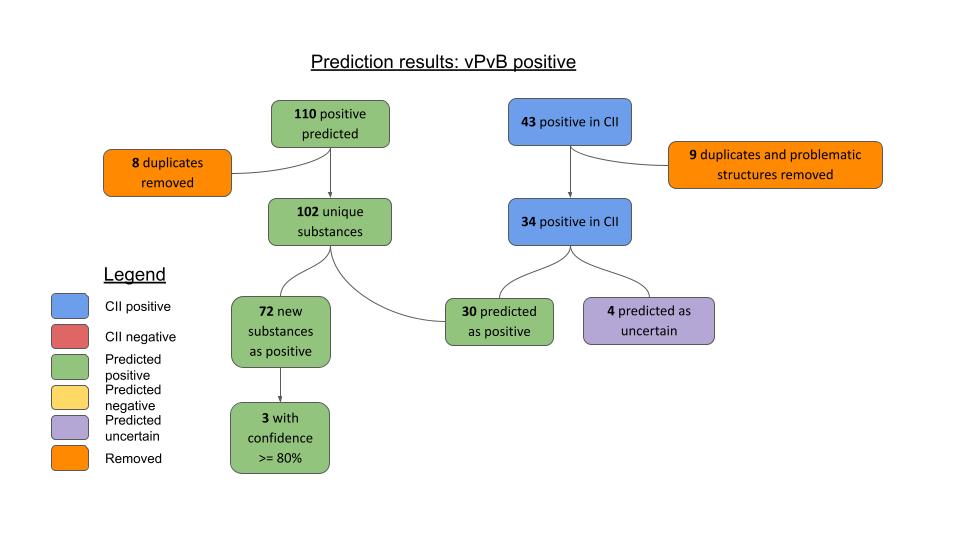

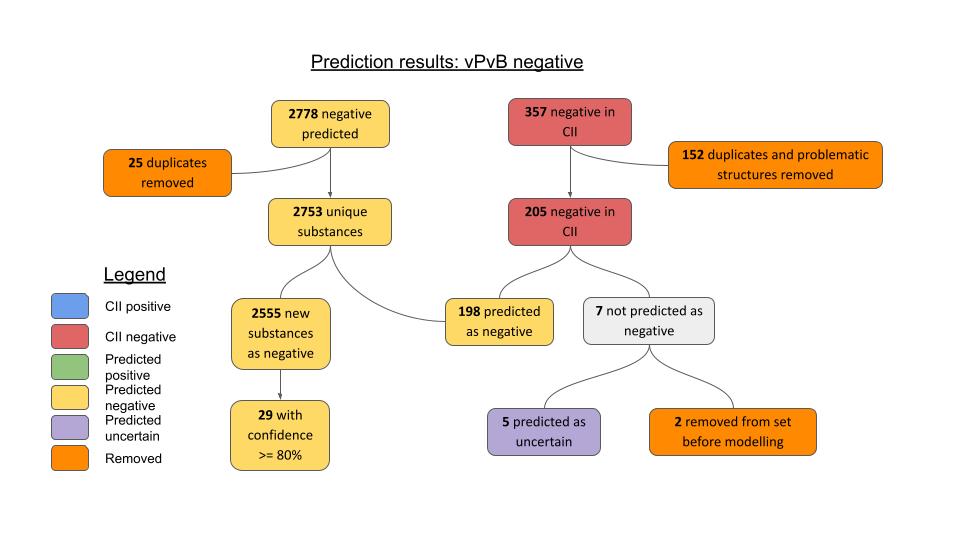

Supplement: Supplementary file 1 — Supplementary file1 (DOCX 2710 KB) [file 204_2023_3459_MOESM1_ESM.docx]
