# Supplementary material for: A continuous in silico learning strategy to identify safety liabilities in compounds used in the leather and textile industry
Source: Arch Toxicol. 2023 Feb 12;97(4):1091–111. doi: 10.1007/s00204-023-03459-7 (PMC10025185; doi:10.1007/s00204-023-03459-7)
Supplement: Supplementary file 2 — Supplementary file2 (PPTX 45 KB) [file 204_2023_3459_MOESM2_ESM.pptx]

## Slide 1
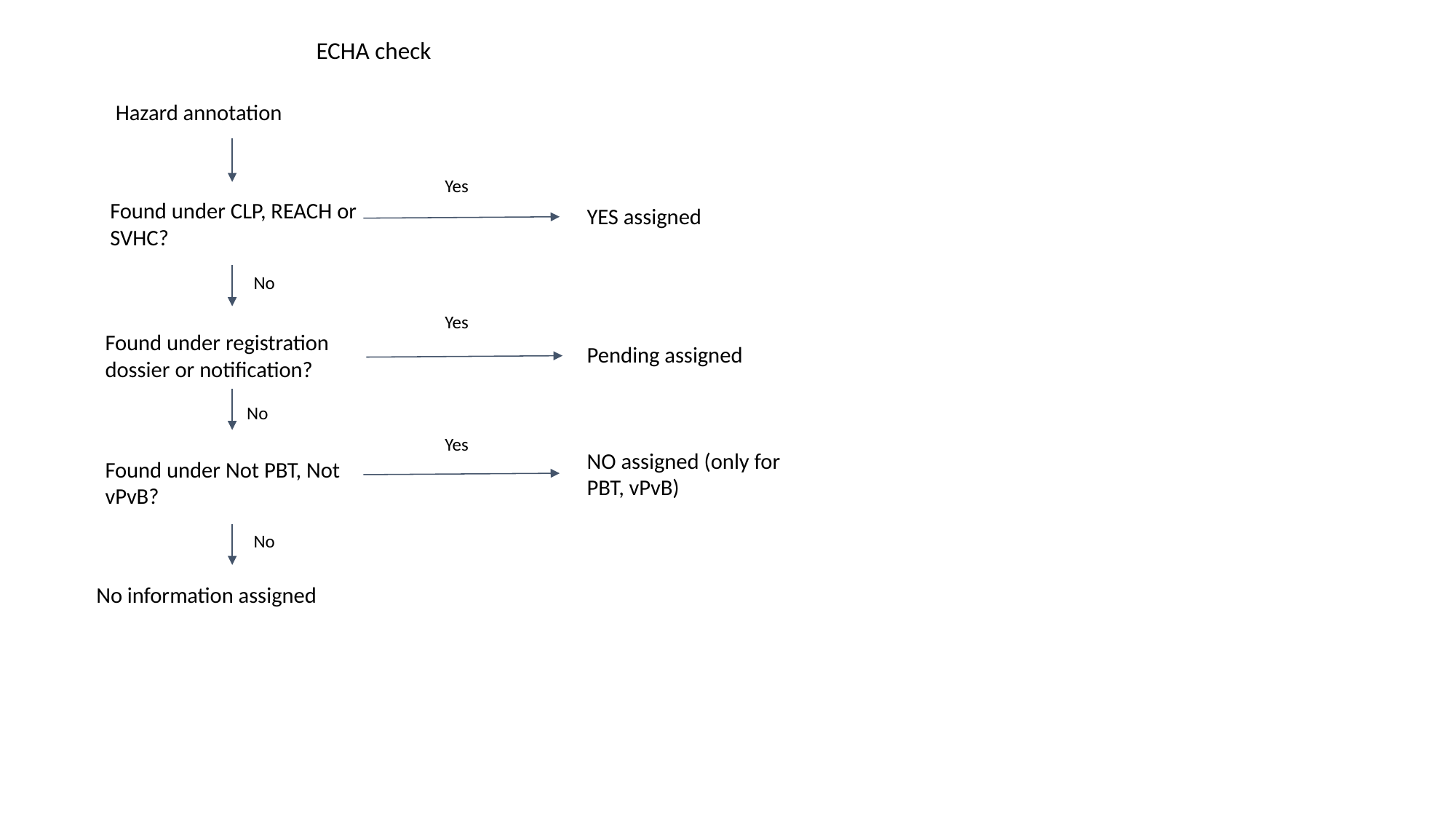

ECHA check
Hazard annotation
Yes
Found under CLP, REACH or SVHC?
YES assigned
No
Yes
Found under registration dossier or notification?
Pending assigned
No
Yes
NO assigned (only for PBT, vPvB)
Found under Not PBT, Not vPvB?
No
No information assigned

## Slide 2
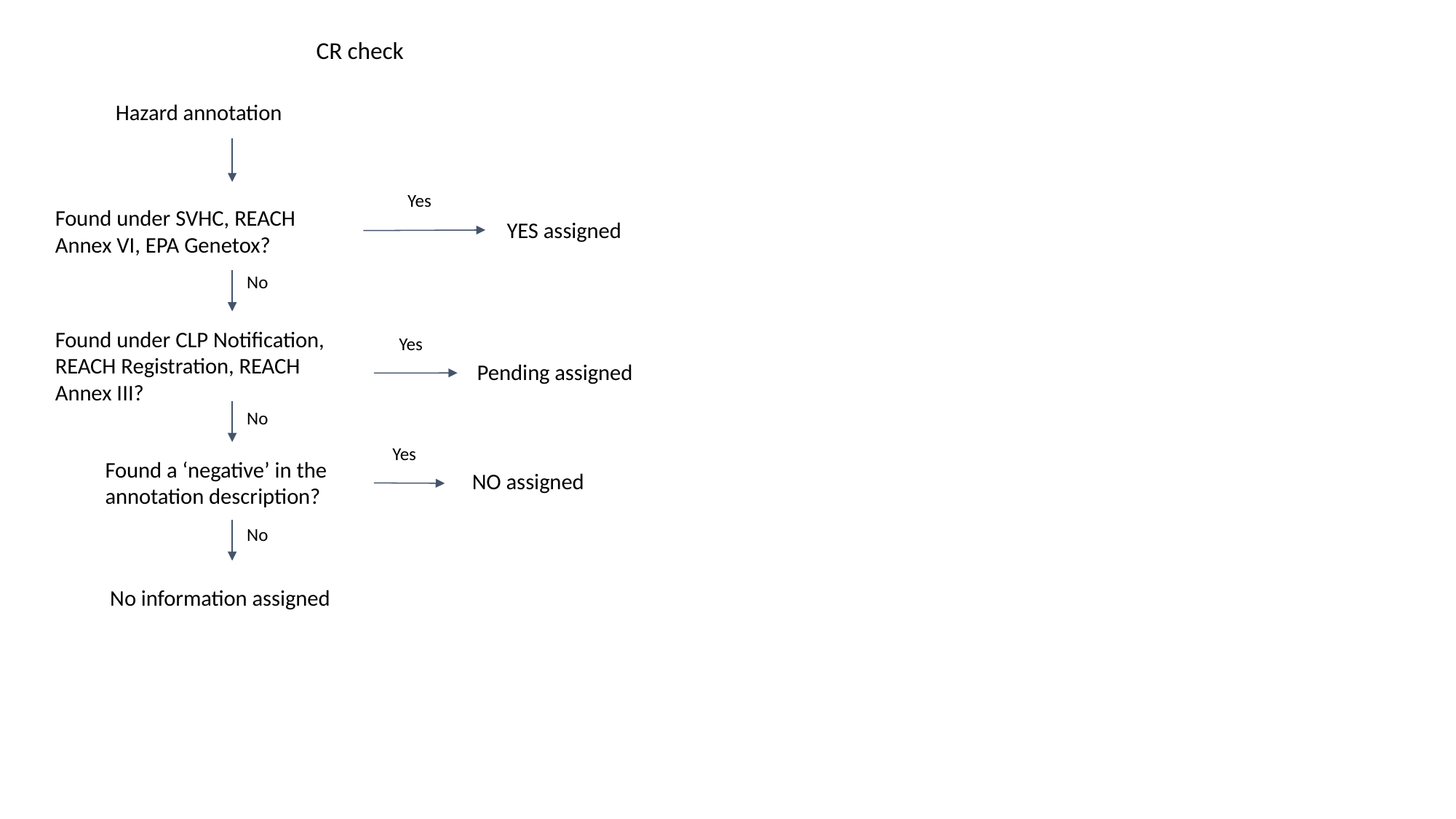

CR check
Hazard annotation
Yes
Found under SVHC, REACH Annex VI, EPA Genetox?
YES assigned
No
Found under CLP Notification, REACH Registration, REACH Annex III?
Yes
Pending assigned
No
Yes
Found a ‘negative’ in the annotation description?
NO assigned
No
No information assigned

## Slide 3
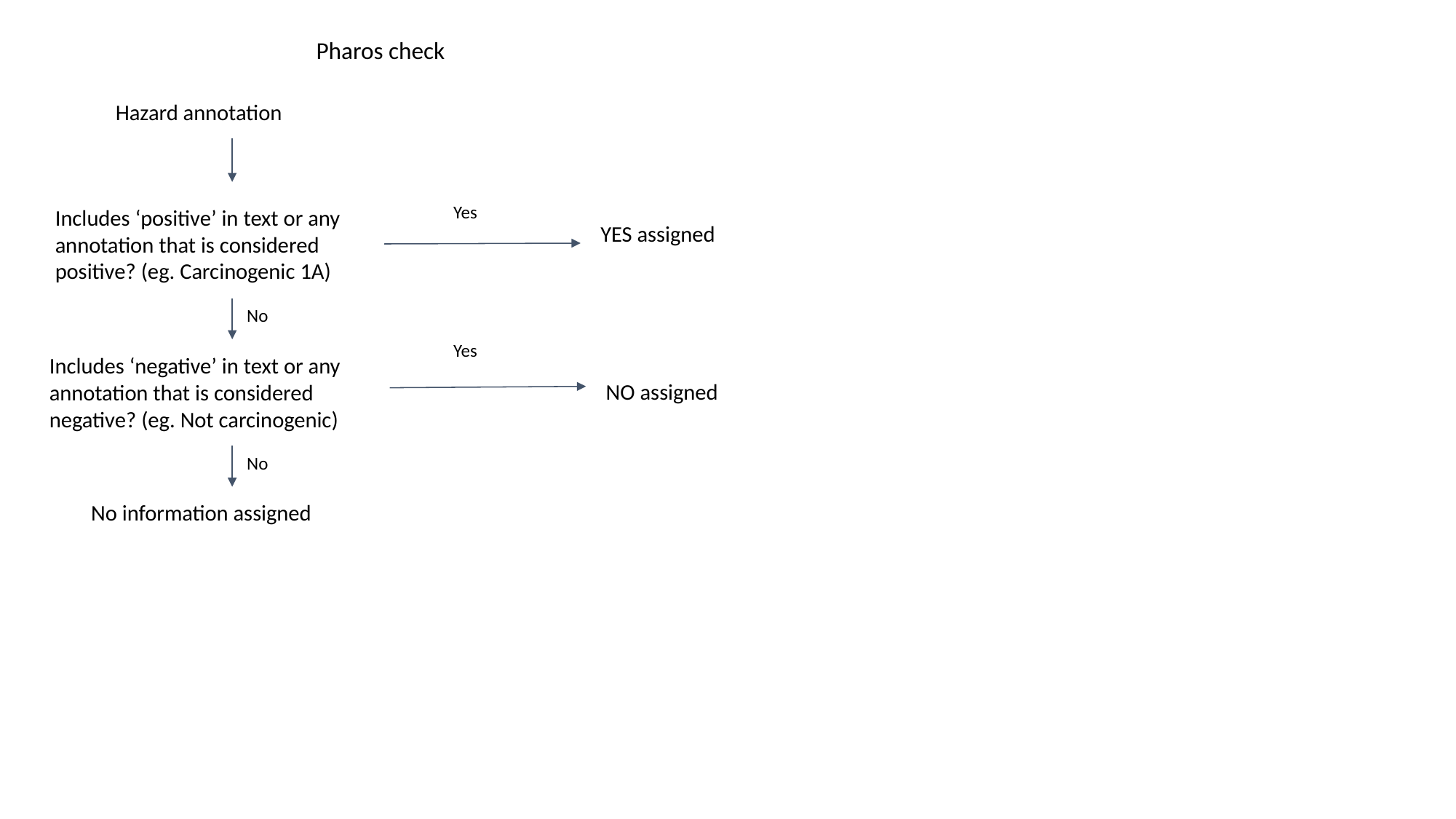

Pharos check
Hazard annotation
Yes
Includes ‘positive’ in text or any annotation that is considered positive? (eg. Carcinogenic 1A)
YES assigned
No
Yes
Includes ‘negative’ in text or any annotation that is considered negative? (eg. Not carcinogenic)
NO assigned
No
No information assigned
