# Supplementary material for: A continuous in silico learning strategy to identify safety liabilities in compounds used in the leather and textile industry
Source: Arch Toxicol. 2023 Feb 12;97(4):1091–111. doi: 10.1007/s00204-023-03459-7 (PMC10025185; doi:10.1007/s00204-023-03459-7)
Supplement: Supplementary file 3 — Supplementary file3 (PPTX 49 KB) [file 204_2023_3459_MOESM3_ESM.pptx]

## Slide 1
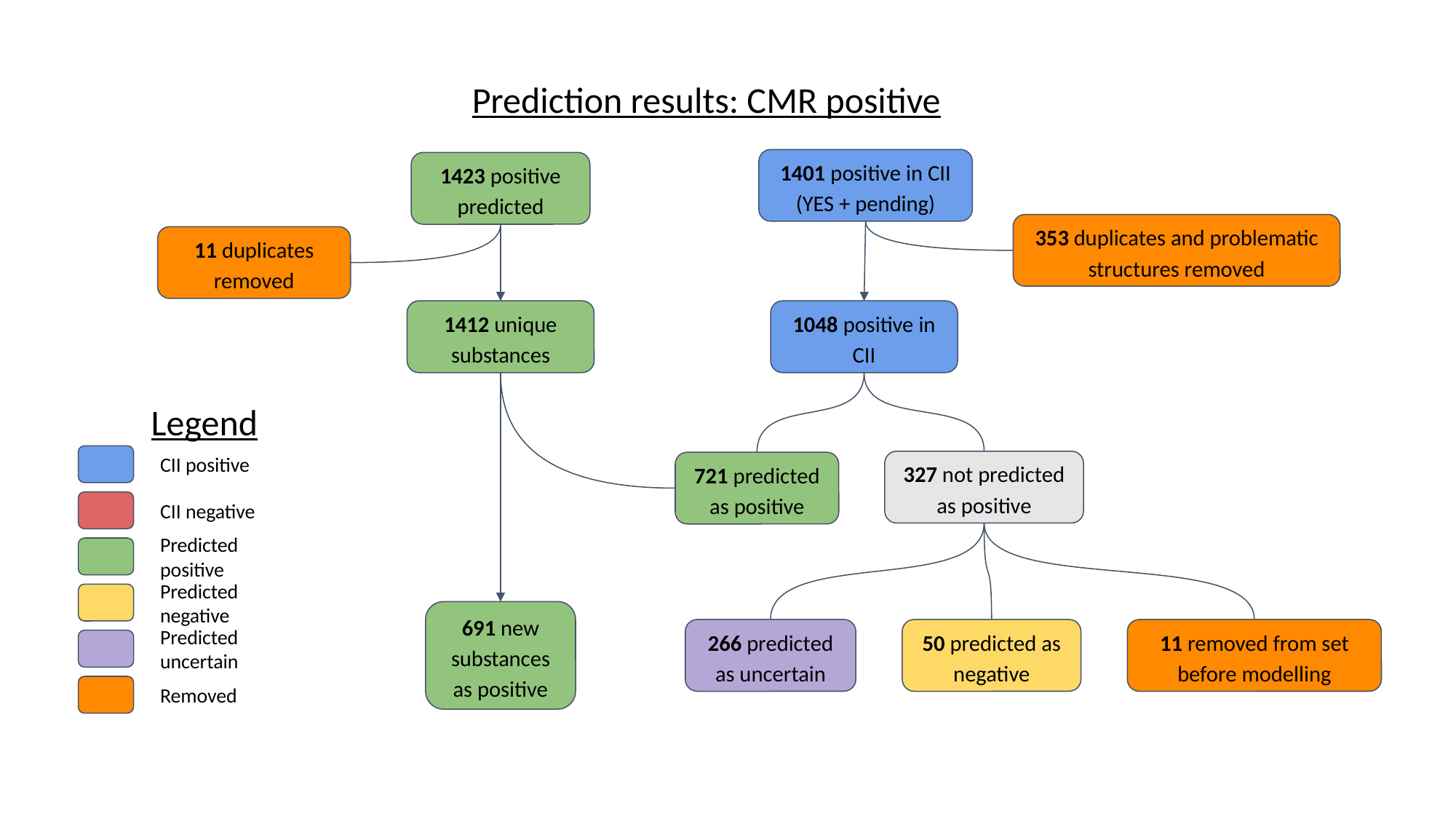

Prediction results: CMR positive
1401 positive in CII (YES + pending)
1423 positive predicted
353 duplicates and problematic structures removed
11 duplicates removed
1412 unique substances
1048 positive in CII
327 not predicted as positive
721 predicted as positive
691 new substances as positive
266 predicted as uncertain
50 predicted as negative
11 removed from set before modelling
Legend
CII positive
CII negative
Predicted positive
Predicted negative
Predicted uncertain
Removed

## Slide 2
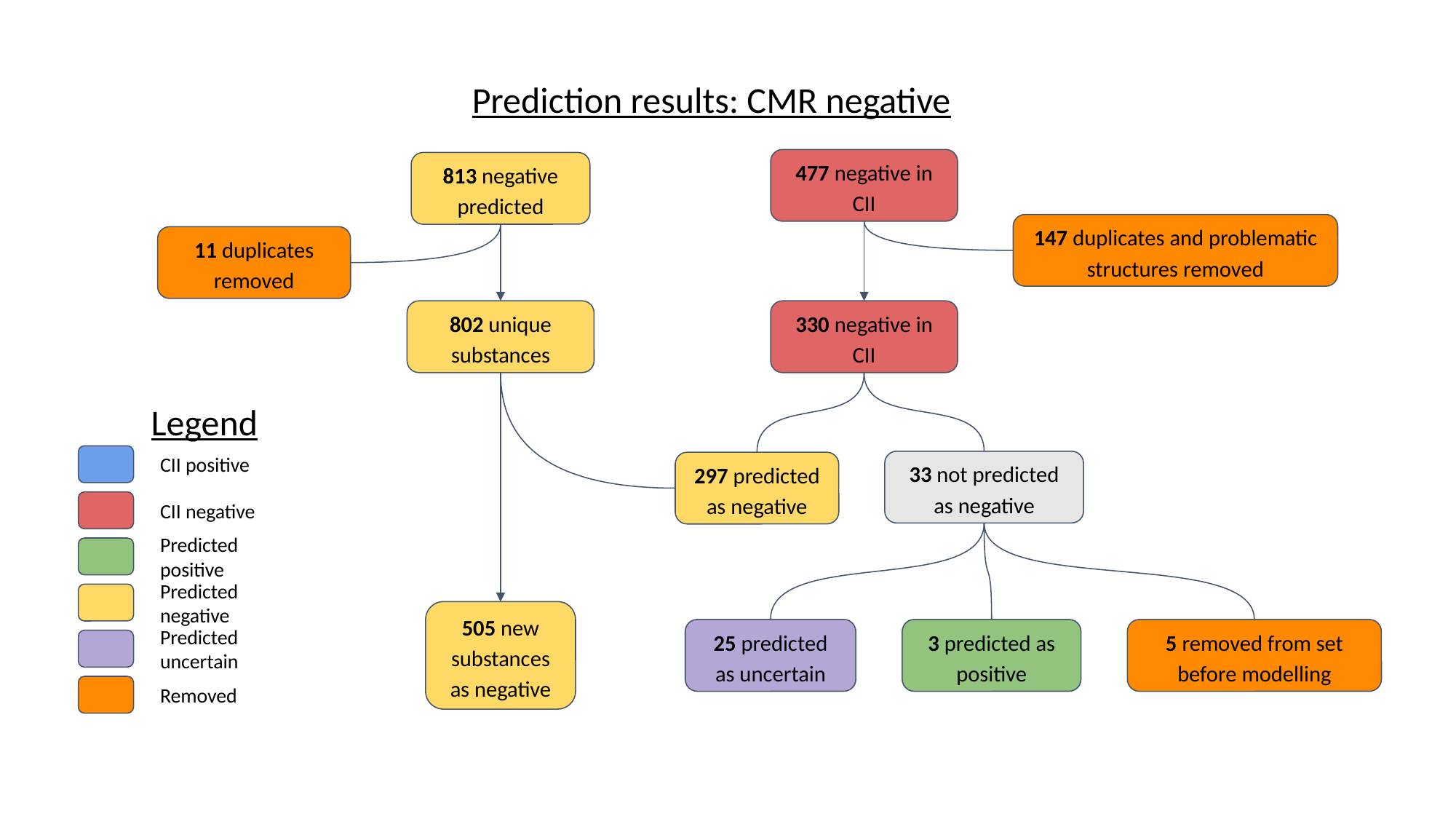

Prediction results: CMR negative
477 negative in CII
813 negative predicted
147 duplicates and problematic structures removed
11 duplicates removed
802 unique substances
330 negative in CII
33 not predicted as negative
297 predicted as negative
505 new substances as negative
25 predicted as uncertain
3 predicted as positive
5 removed from set before modelling
Legend
CII positive
CII negative
Predicted positive
Predicted negative
Predicted uncertain
Removed
