# Supplementary material for: A continuous in silico learning strategy to identify safety liabilities in compounds used in the leather and textile industry
Source: Arch Toxicol. 2023 Feb 12;97(4):1091–111. doi: 10.1007/s00204-023-03459-7 (PMC10025185; doi:10.1007/s00204-023-03459-7)
Supplement: Supplementary file 4 — Supplementary file4 (PPTX 47 KB) [file 204_2023_3459_MOESM4_ESM.pptx]

## Slide 1
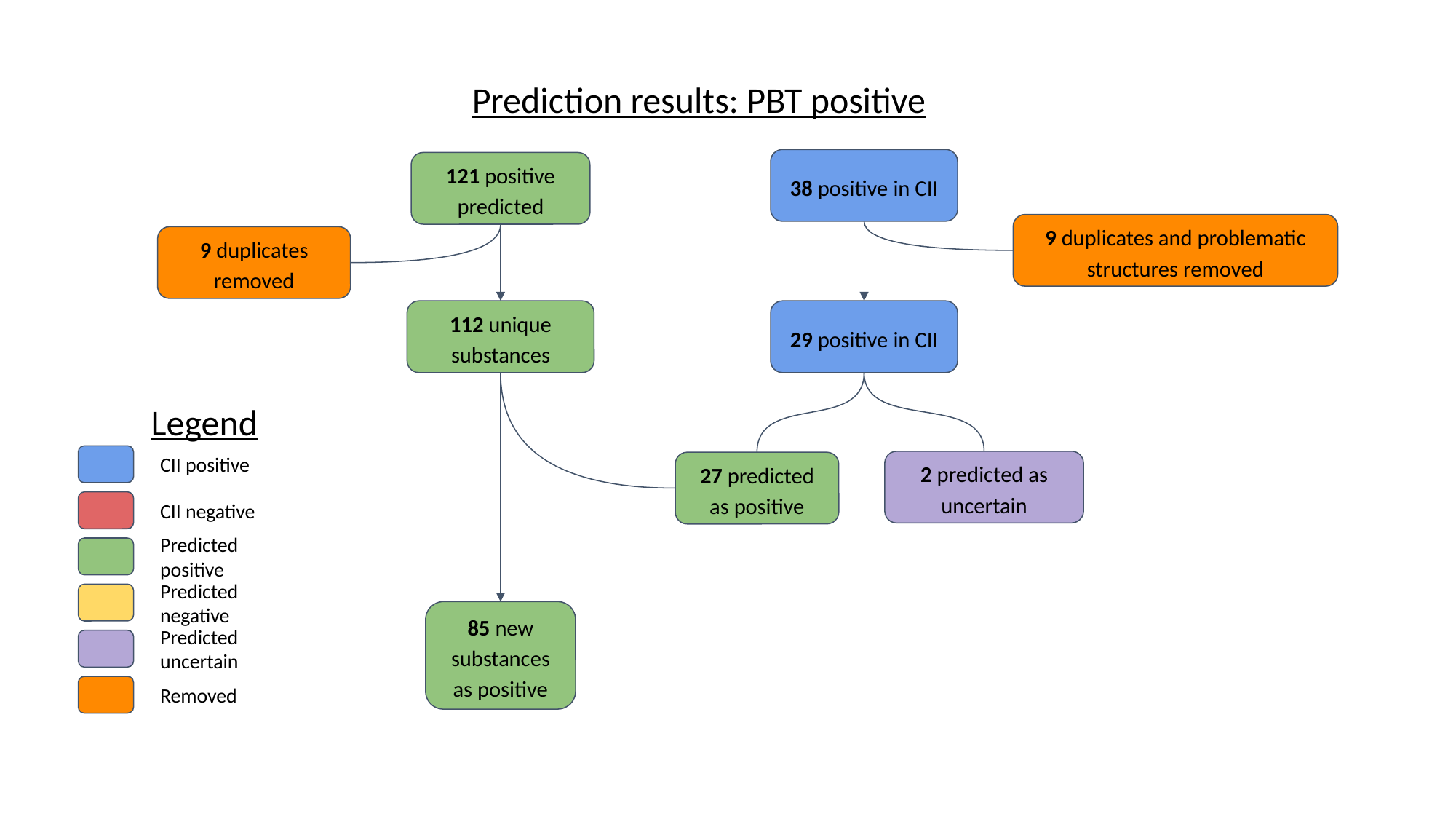

Prediction results: PBT positive
38 positive in CII
121 positive predicted
9 duplicates and problematic structures removed
9 duplicates removed
112 unique substances
29 positive in CII
2 predicted as uncertain
27 predicted as positive
85 new substances as positive
Legend
CII positive
CII negative
Predicted positive
Predicted negative
Predicted uncertain
Removed

## Slide 2
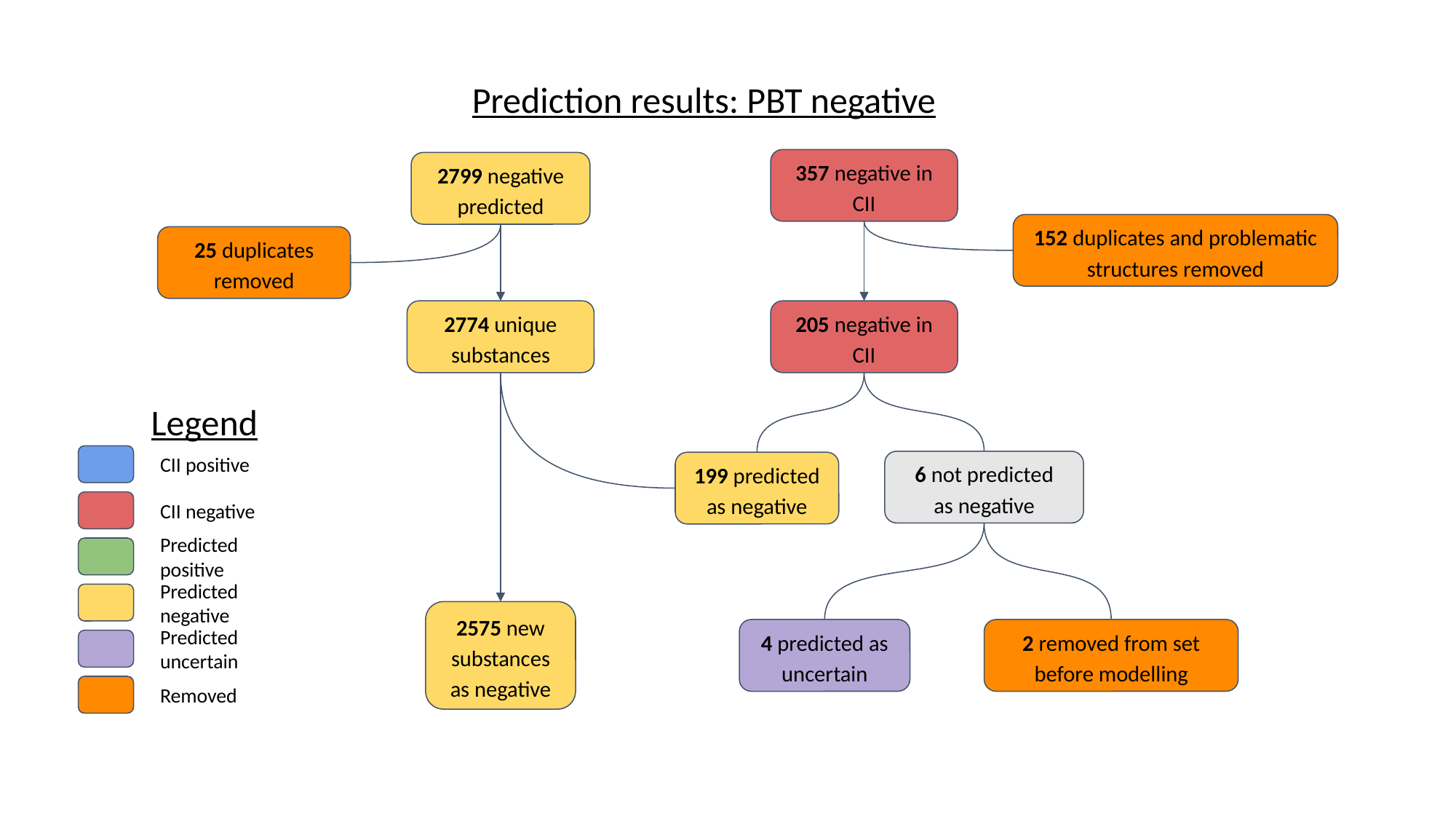

Prediction results: PBT negative
357 negative in CII
2799 negative predicted
152 duplicates and problematic structures removed
25 duplicates removed
2774 unique substances
205 negative in CII
6 not predicted as negative
199 predicted as negative
2575 new substances as negative
Legend
CII positive
CII negative
Predicted positive
Predicted negative
Predicted uncertain
Removed
4 predicted as uncertain
2 removed from set before modelling
