# Supplementary material for: A continuous in silico learning strategy to identify safety liabilities in compounds used in the leather and textile industry
Source: Arch Toxicol. 2023 Feb 12;97(4):1091–111. doi: 10.1007/s00204-023-03459-7 (PMC10025185; doi:10.1007/s00204-023-03459-7)
Supplement: Supplementary file 5 — Supplementary file5 (PPTX 47 KB) [file 204_2023_3459_MOESM5_ESM.pptx]

## Slide 1
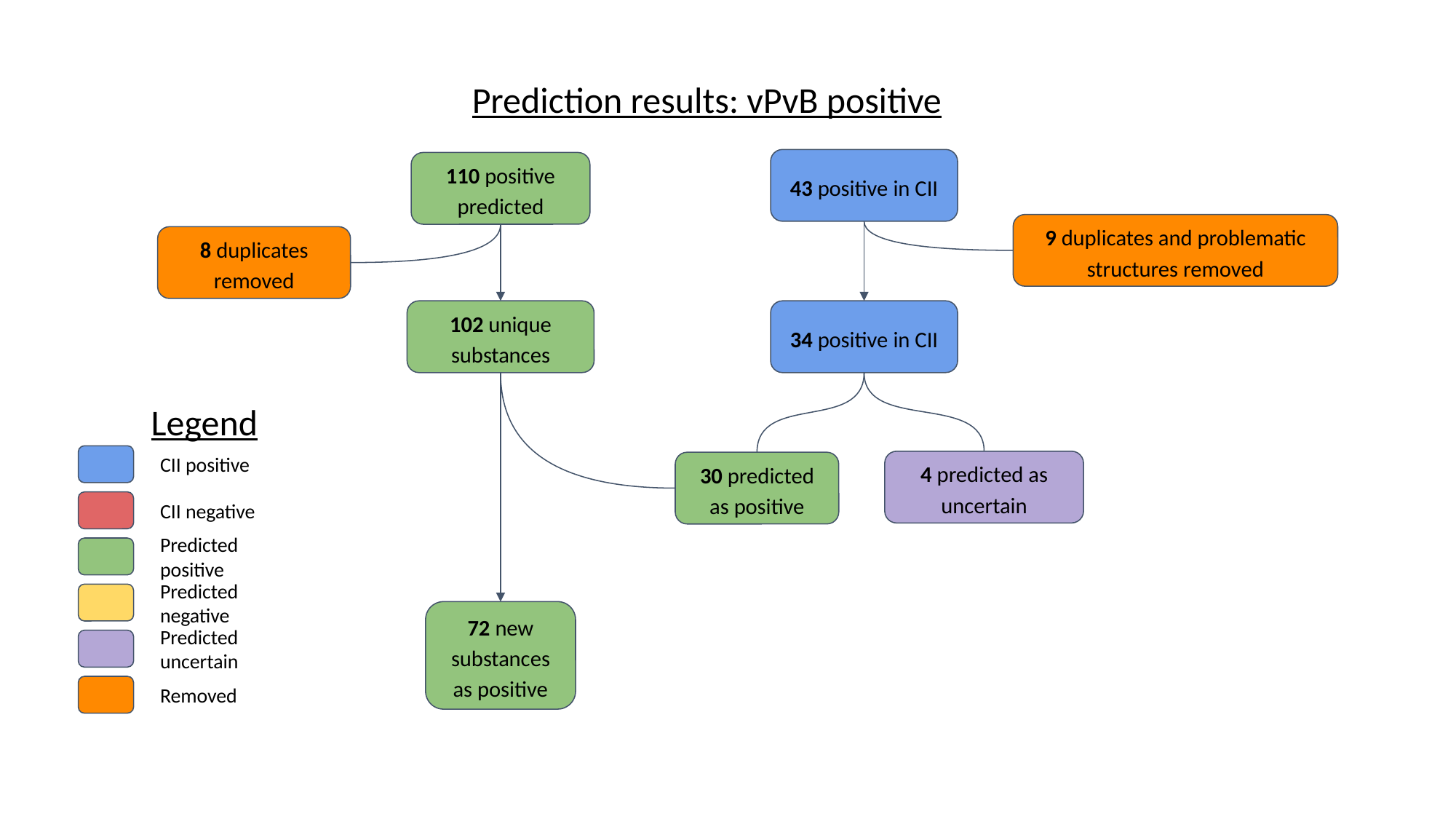

Prediction results: vPvB positive
43 positive in CII
110 positive predicted
9 duplicates and problematic structures removed
8 duplicates removed
102 unique substances
34 positive in CII
4 predicted as uncertain
30 predicted as positive
72 new substances as positive
Legend
CII positive
CII negative
Predicted positive
Predicted negative
Predicted uncertain
Removed

## Slide 2
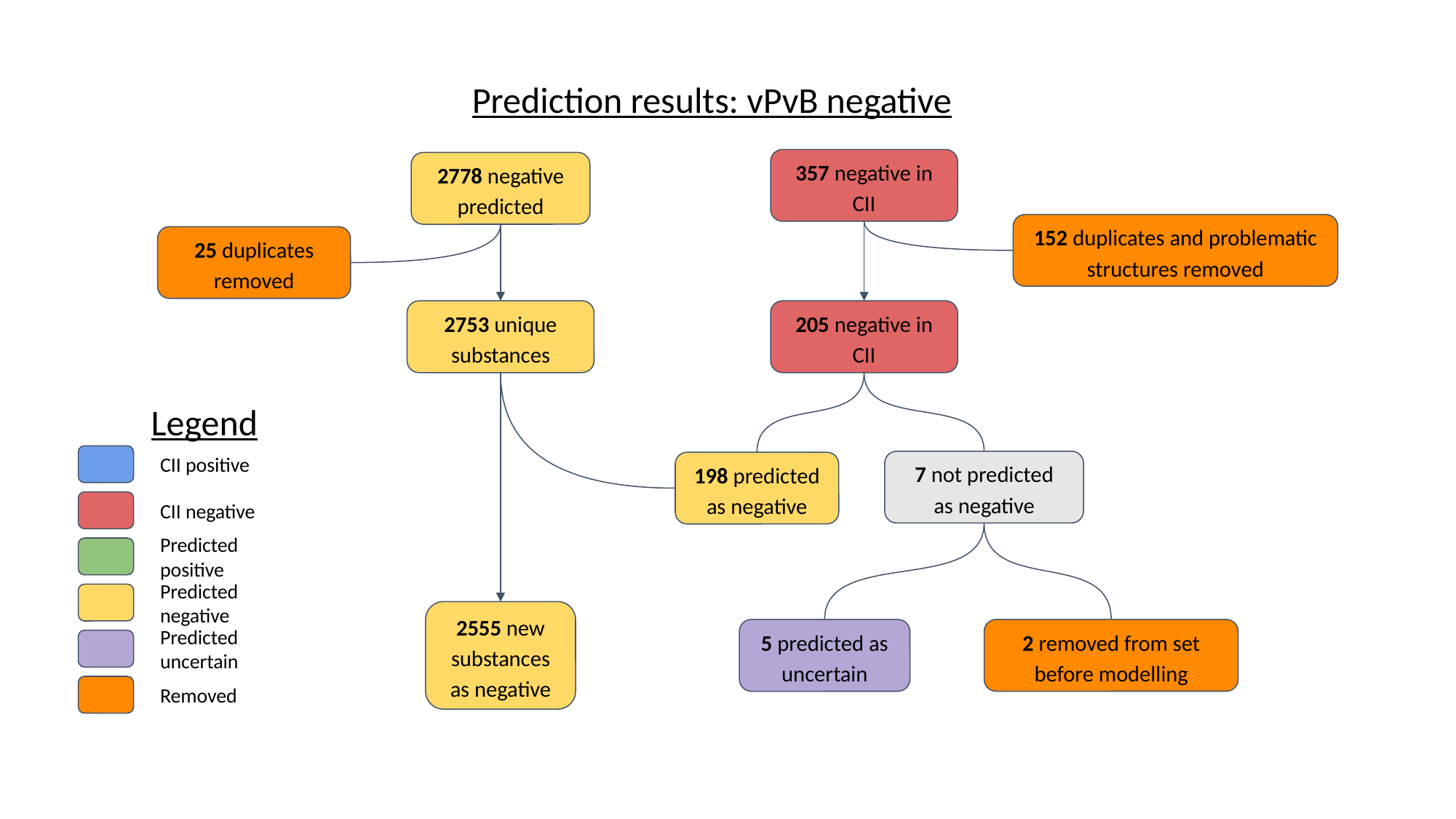

Prediction results: vPvB negative
357 negative in CII
2778 negative predicted
152 duplicates and problematic structures removed
25 duplicates removed
2753 unique substances
205 negative in CII
7 not predicted as negative
198 predicted as negative
2555 new substances as negative
Legend
CII positive
CII negative
Predicted positive
Predicted negative
Predicted uncertain
Removed
5 predicted as uncertain
2 removed from set before modelling
